# Symbiont Acquisition Strategies in Post‐Settlement Stages of Two Co‐Occurring Deep‐Sea *Rimicaris* Shrimp

**DOI:** 10.1002/ece3.70369

**Published:** 2024-11-19

**Authors:** Marion Guéganton, Pierre Methou, Johanne Aubé, Cyril Noël, Ouafae Rouxel, Valérie Cueff‐Gauchard, Nicolas Gayet, Lucile Durand, Florence Pradillon, Marie‐Anne Cambon‐Bonavita

**Affiliations:** ^1^ Univ Brest, Ifremer, CNRS, Unite Biologie des Environnements Extrêmes marins Profonds Plouzane France; ^2^ Ifremer, IRSI, SeBiMER Service de Bioinformatique de l'Ifremer Plouzané France

**Keywords:** acquisition, FISH, hydrothermal vent, metabarcoding, microscopy, symbiosis, symbiotic transmission

## Abstract

At deep‐sea hydrothermal vents, deprived of light, most living communities are fueled by chemosynthetic microorganisms. These can form symbiotic associations with metazoan hosts, which are then called holobionts. Among these, two endemic co‐occurring shrimp of the Mid‐Atlantic Ridge (MAR), *Rimicaris exoculata* and *Rimicaris chacei* are colonized by dense and diversified chemosynthetic symbiotic communities in their cephalothoracic cavity and their digestive system. Although both shrimp harbor similar communities, they exhibit widely different population densities, distribution patterns at small scale and diet, as well as differences in post‐settlement morphological modifications leading to the adult stage. These contrasting biological traits may be linked to their symbiotic development success. Consequently, key questions related to the acquisition of the symbiotic communities and the development of the three symbiotic organs are still open. Here we examined symbiotic development in juveniles of *R. exoculata* and *R. chacei* from TAG and Snake Pit using 16S metabarcoding to identify which symbiotic lineages are present at each juvenile stage. In addition, we highlighted the abundance and distribution of microorganisms at each stage using Fluorescence in situ Hybridization (FISH) and Scanning Electron Microscopy (SEM). For the first time, *Candidatus* Microvillispirillaceae family with *Candidatus* Rimicarispirillum spp. (midgut tube), *Candidatus* Foregutplasma rimicarensis and *Candidatus* BG2‐rimicarensis (foregut) were identified in late juvenile stages. However, these lineages were absent in early juvenile stages, which coincides for the midgut tube with our observations of an immature tissue, devoid of microvilli. Conversely, symbiotic lineages from the cephalothoracic cavity were present from the earliest juvenile stages of both species and their overall diversities were similar to those of adults. These results suggest different symbiont acquisition dynamics between the cephalothoracic cavity and the digestive system, which may also involve distinct transmission mechanisms.

## Graphical abstract

*Rimicaris exoculata* and *Rimicaris chacei* are two shrimp from hydrothermal vents of the Mid‐Atlantic Ridge which host symbiotic communities in their cephalothoracic cavity and their digestive system. Our study explores colonization dynamics and symbiont acquisition pathways of these two symbioses along the metamorphosis phase between juvenile and adult stages of these shrimps. Altogether, our analyses suggest different symbiont acquisition dynamics between the cephalothoracic cavity and the digestive system, which may also involve distinct transmission mechanisms.

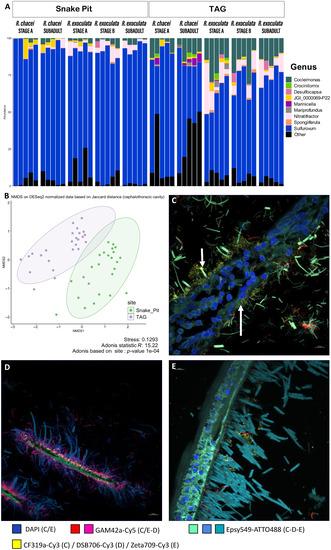

## 1 Introduction

Most hydrothermal vents occur at depths deprived of light where photosynthesis is not possible. In these habitats, chemosynthesis is the main biosynthetic process, performed by microorganisms, living free or in association with metazoan hosts (Kouris et al. 2007; Dubilier, Bergin, and Lott 2008; Sogin et al. 2021). These associations between host and microbial symbionts, now called holobionts (Zilber‐Rosenberg and Rosenberg 2008), dominate vent communities. They usually involve large engineer species like *Bathymodiolus* spp. mussels or *Riftia* tubeworms (Nussbaumer, Fisher, and Bright 2006; Duperron et al. 2006, 2008; Duperron 2010), which have developed diversified symbioses in specific organs. To ensure the maintenance of their symbiotic relationship over successive generations, symbionts are transmitted horizontally (Nussbaumer, Fisher, and Bright 2006; Bright and Bulgheresi 2010; Wentrup et al. 2014). Cases of vertical transmission (directly from the parents) or mixed mode of transmission (both vertical and horizontal) also exist respectively for *Calyptogena* clams and for the scaly‐foot snail (Ikuta et al. 2016; Lan et al. 2022). These acquisition phases often go with marked changes in the external and/or internal anatomy of their hosts (Nussbaumer, Fisher, and Bright 2006; Chen et al. 2018; Franke et al. 2021), which can be triggered by the symbionts themselves (Chun et al. 2008). Hence, symbionts are now increasingly recognized as fundamental actors of their host developmental processes in most metazoan phyla (Carrier and Bosch 2022).

The shrimp *Rimicaris exoculata* and *Rimicaris chacei* are symbiotic species co‐occurring at active vents along the Mid‐Atlantic Ridge (MAR) (Williams and Rona 1986; Zbinden and Cambon Bonavita 2020). *R. exoculata* lives in dense aggregations (up to 3000 individuals per m^2^) covering the substratum of active black smoker walls (Segonzac, de Saint Laurent, and Casanova 1993; Hernández‐Ávila et al. 2022; Methou et al. 2022). This shrimp can be exposed to quite high temperatures (10°C–30°C) and to diverse chemical compounds (Schmidt et al. 2008a, 2008b; Methou et al. 2022). *R. chacei* seems to be less abundant, and lives in different ecological niches, usually with lower hydrothermal influence (Hernández‐Ávila et al. 2022; Methou et al. 2022).

In adulthood, both species have distinct morphologies. *R. exoculata* exhibits a laterally inflated cephalothoracic cavity enclosing the two first pairs of pereiopods (including chelipeds) and hypertrophied mouthparts (such as scaphognathites and exopodites) (Van Dover et al. 1988; Segonzac, de Saint Laurent, and Casanova 1993; Komai and Segonzac 2008). On the contrary, the chelipeds of *R. chacei* remain free and functional as its cephalothoracic cavity and mouthparts are not as hypertrophied as *R. exoculata* (Casanova, Brunet, and Segonzac 1993; Segonzac, de Saint Laurent, and Casanova 1993). Despite these morphological differences, both species harbor dense bacterial communities in their cephalothoracic cavity, colonizing the inner side of the branchiostegites as well as the scaphognathites and the exopodites and their setae (Apremont et al. 2018). In both *R. exoculata* and *R. chacei* adults, symbiotic communities of the cephalothoracic cavity are mainly composed of *Campylobacteria* (*Campylobacterales* order, mostly *Sulfurovum* spp. and *Nitrtifractor* spp.), then *Gammaproteobacteria* (mostly *Cocloeimonas* spp.), followed by minor proportions of *Alphaproteobacteria* (mostly *Marinosulfonomonas* spp.), *Desulfobulbia* (*Desulfobulbales* order, mostly *Desulfocapsa* spp.), *Zetaproteobacteria* (mostly *Candidatus Ghiorsea cryta* and *Candidatus Ghiorsea rimicarensis*), Patescibacteria (GCA‐2747955 family) and *Bacteroidia* (mostly *Crocinitomix* spp. and *Spongiiferula* spp.) (Zbinden et al. 2008; Petersen et al. 2010; Hügler, Gärtner, and Imhoff 2010; Guri et al. 2012; Jan et al. 2014; Apremont et al. 2018; Jiang et al. 2020; Cambon‐Bonavita et al. 2021). In *R. exoculata*, these ectosymbiotic communities are renewed every 10 days after each molt event (Corbari et al. 2008a, 2008b) and play a major trophic role for their host, as evidenced by their isotopic ratios (Polz et al. 1998; Gebruk et al. 2000; Methou et al. 2020). This was confirmed by experiments with radiolabeled inorganic carbon showing a direct transfer of the ^14^C incorporated by the symbionts to the host through the cuticle of the cephalothorax (Ponsard et al. 2013). Conversely, *R. chacei* appears to have a mixotrophic feeding behavior (Gebruk et al. 2000; Methou et al. 2020). Indeed, its alimentary bolus contains some minerals, cuticle debris and organic waste (Casanova, Brunet, and Segonzac 1993; Apremont et al. 2018). This cuticle and the organic matter, according to isotopic analyses (nitrogen), may come from *R. exoculata* juveniles, which would then be potential prey for adult *R. chacei* (observed in situ by Gebruk et al. 2000). This suggests that *R. chacei* has a trophic behavior similar to that of predators or scavengers. The energy intake of *R. chacei* is also thought to be symbiotic in origin. Studies of isotopic ratios of sulfur δ^34^S and carbon δ^13^C (Methou et al. 2020), coupled with other data showed a trend towards a mixed trophic diet based on bacteriotrophy, symbiosis, predation and scratching (Casanova, Brunet, and Segonzac 1993; Apremont et al. 2018).

As in other decapods, the digestive system of both *R. exoculata* and *R. chacei* is divided in three distinct regions: the foregut composed of the esophagus and the stomach, the midgut that comprises the hepatopancreas and the midgut tube, and the hindgut that is the terminal excretion zone (Vogt 2021). For both species, the foregut is a complex filtering structure made of several plates and setae (Guéganton et al. 2022). Contrary to the foregut and the hindgut which have an ectodermic origin, the midgut tube is devoid of a cuticle (endodermic origin) and is thus not subjected to molt (Vogt 2021). The foregut and hindgut of *R. exoculata* are reduced whereas the midgut tube is very long (Komai and Segonzac 2008; Durand et al. 2009; Guéganton et al. 2022; Methou et al. 2023b). On the contrary, the digestive system of *R. chacei* is more similar to that of other caridean shrimp (Segonzac, de Saint Laurent, and Casanova 1993; Komai and Segonzac 2008; Apremont et al. 2018; Methou et al. 2023b) with a relatively large stomach, in agreement with its mixotrophic behavior.

Like the cephalothoracic cavity, the digestive system of *R. exoculata* and *R. chacei* hosts symbiotic microorganisms (Durand et al. 2009, 2015; Apremont et al. 2018). Two distinct communities were identified: the first one is located in the foregut and consists mainly of *Candidatus* Foregutplasma rimicarensis and *Candidatus* BG2_rimicarensis (Aubé et al. 2022). In adult shrimp, these symbionts have been observed on the setae of the esophagus and in the pyloric chamber of the stomach (Guéganton et al. 2022). In the midgut tube, microbial communities consist mainly of *Candidatus* Microvillispirillaceae with mostly *Candidatus* Rimicarispirillum spp. (Aubé et al. 2022) that form long thin “spaghetti‐like” bacteria cells (Guéganton et al. 2022). These are inserted between the microvilli of the epithelial cells, colonizing the ectoperitrophic space (Guéganton et al. 2022), which is recognized as “sterile” in several other crustaceans (Martin et al. 2020). *Candidatus* Rimicarispirillum spp. are not subject to molt and their growth is believed to be under host control as they exhibit no cell division (Durand et al. 2009; Apremont et al. 2018), while having all the genes for chromosome replication and cell division (Aubé et al. 2022).

While these three symbioses located in three ‘symbiotic organs’ (cephalothoracic chamber, foregut and midgut tube with the hindgut respectively) are well characterized in *Rimicaris* adults, less is known about these bacterial partners at juvenile stages (Guri et al. 2012; Cowart et al. 2017), particularly for *R. chacei*. Juveniles are easily recognizable from adults because of their red/orange color, which is due to lipid storage (Pond et al. 2000; Methou et al. 2020). Both species are relatively similar at these early stages, leading to potential misidentifications, but the taxonomy of early stages was recently revised, clarifying the different juvenile stages of each species (Methou et al. 2020). In their environment, juveniles of *R. chacei* live in nurseries and are separated from their adults, whereas juveniles of *R. exoculata* gather in patches adjacent to adult assemblages (Hernández‐Ávila et al. 2022; Methou et al. 2022). Whereas *R. exoculata* adults were in higher numbers than juveniles, juveniles of *R. chacei* showed a high abundance contrasting with a relatively low number of their adults (Methou et al. 2022). This limited number of *R. chacei* adults could be related to a collapse of the population during recruitment. Such differences in the demography of the two *Rimicaris* species at MAR vent sites might stem from differences in the niches they occupy and/or the symbiosis development in juveniles following a settlement with metamorphoses differing according to symbiont colonization level (Methou et al. 2023b).

However, the timing of symbiont acquisition and the colonization dynamic of each hosting organ remain unclear. Although a vertical transmission was suggested for midgut symbionts (Durand et al. 2015), these lineages could not be detected in egg broods of *R. exoculata* (Guri et al. 2012; Cowart et al. 2017; Methou et al. 2019). On the other hand, digestive symbionts could not be found in the environment of the shrimp either (Hügler, Gärtner, and Imhoff 2010; Flores et al. 2011). *Rimicaris* broods nevertheless exhibit bacterial communities developing on the envelope of eggs with similar lineages to those found in the cephalothoracic cavity (Methou et al. 2019). A transmission of these symbionts to larval stages by ingestion/scraping of the egg envelope is unlikely as *Rimicaris* larvae have undeveloped buccal organs preventing the ingestion of materials upon hatching (Hernández‐Ávila, Cambon‐Bonavita, and Pradillon 2015). Moreover, the nutrition of juveniles of both *Rimicaris* species before recruitment is largely based on photosynthetic‐derived organic matter and gradually shifts towards a chemosynthetic diet during metamorphosis (Methou et al. 2020). This trophic shift may reflect the development of symbioses suggesting that recruitment could be a key phase of symbiont acquisition for these shrimp.

Therefore, we focused here on the symbiont acquisition in each of the three symbiotic organs in the juvenile stages of *R. exoculata* and *R. chacei* from two vent sites along the MAR (TAG and Snake Pit) using a combination of microscopy (Fluorescent in situ Hybridization [FISH]) and Scanning Electron Microscopy (SEM) and 16S rRNA metabarcoding to address the following questions: (1) Which are the earliest juvenile stages in which each of the known adult symbiotic lineages can be detected? (2) What are the dynamics of the different symbiotic communities throughout juvenile development? (3) Does these acquisition timing and dynamics vary between host species and vent sites? (4) Is the establishment of symbiotic communities linked to anatomical changes of the host organs?

## 2 Materials and Methods

### 2.1 Sample Collections

Samples were collected at two vent fields along the MAR: TAG (26°08′ N—44°49′ W, 3660 m depth) and Snake Pit (23°23′ N—44°58′ W, 3480 m depth) during the BICOSE cruise (10 January to 11 February 2014, DOI https://doi.org/10.17600/14000100), the HERMINE cruise (16 March to 27 April 2017, DOI https://doi.org/10.17600/17000200) and the BICOSE2 cruise (26 January to 10 March 2018, DOI https://doi.org/10.17600/18000004). The different specimens were caught in shrimp aggregations or nurseries using the suction sampler of the HOV (Human Occupied Vehicle) Nautile, or the ROV (Remotely Operated Vehicle) VICTOR 6000, operated from the R/V *Pourquoi pas*. Once on board, some shrimp were immediately dissected under sterile conditions and tissues were fixed for FISH studies in a 3% formalin seawater solution for 3 h to keep cell integrity. Other specimens were fixed without dissection for whole individual observations. Samples were then rinsed with a phosphate‐buffered saline solution (PBS) and stored in a PBS/Ethanol (1:1) solution at −20°C (Durand et al. 2009). Tissues of some specimens were fixed in a 2.5% glutaraldehyde solution (16 h at 4°C), and then rinsed and stored at 4°C in buffered filtered seawater containing a biocide (NaN_3_ at 0.44 g/L) to avoid bacterial development until use for scanning electron microscopy (SEM) observations. Other shrimp specimens were frozen at −80°C for later dissections at the laboratory for DNA extraction or fixation for FISH (Appendix S1).

### 2.2 DNA Extraction

Juvenile specimens of *R. exoculata* and *R. chacei* were sorted and identified on board following criteria defined by Methou et al. (2020). Briefly, their identification was based on morphological features of the cephalothorax (shape of their rostrum and carapace, eye fusion level; see table 1 in Methou et al. (2020) for additional details). At the laboratory, we defrosted and aseptically dissected on ice 50 specimens of *R. chacei* and *R. exoculata* from TAG and Snake Pit (stages A, B, and subadults) under a binocular microscope (Appendices S1 and S2) To avoid contamination, the dissecting tools were rinsed in ethanol 96°, then DNA away, Milli‐Q Water and then ethanol 96°, between each organ dissection. For each specimen used in the metabarcoding analysis, species identity was further confirmed through *COI*—*cytochrome oxidase I*—sequencing using DNA extracted from pleopods. For prokaryotic metabarcoding, the midgut tube, foregut, gills, branchiostegites, scaphognathites, and exopodites were dissected (the three last were pooled together for DNA extraction and metabarcoding) (Appendix S2). Pleopod DNA was extracted using the E.Z.N.A Tissue DNA Kit (Omega BIO‐TEK), following the instructions of the manufacturer. DNA was eluted in 100 μL (total) Elution Buffer and then stored at −20°C. DNA for bacterial metabarcoding was extracted from dissected tissues using the Kit Genomic DNA from Soil: NucleoSpin Soil kit (Macherey‐Nagel) following instructions of the manufacturer, using SL2 lysis buffer. DNA was eluted in 30 or 50 μL (respectively digestive system tissues or cephalothoracic tissues) SE Buffer and then stored at −20°C.

### 2.3 COI Sequencing

DNA amplification, using a Gene‐Amp PCR System 9700 (Applied Biosystems, Forster City, CA), was performed with the specific probes Cari‐COI‐1F (5′‐GCAGTCTRGYGTCTTAATTTCCAC‐3′) and Cari‐COI‐1R (5′‐GCTTCTTTTTTACCRGATTCTTGTC‐3′) (Hernández‐Ávila, 2016) which produce a 891 bp fragment (Appendix S3). The PCR products were sequenced (Sanger Sequencing) at Eurofins Genomics GmbH. Sequences were aligned with the MUSCLE (MUltiple Sequence Comparison by Log‐Expectation) algorithm (Edgar, 2004) using Geneious software v9 (Kearse et al. 2012) and were compared to three *COI* sequences of *R. exoculata* adults (MT270775, MT270774 et MT270776) and three *COI* sequences of *R. chacei* adults (MT270739, MT270748 et MT270708) using the BLAST search program within the NCBI GenBank database (Altschul et al. 1990).

### 2.4 Bacterial 16S rRNA Gene Amplifications

Amplification of the bacterial 16S rRNA gene was performed with the universal primers Whoi341 (5′‐CTTTCCCTACACGACGCTCTTCCGATCTCCTACGGGNGGCWGCAG‐3′) and Whoi785 (5′‐GGAGTTCAGACGTGTGCTCTTCCGATCTGACTACHVGGGTATCTAATCC‐3′) with adapter (Herlemann et al. 2011), which targets the V3—V4 region of the bacterial 16S rRNA gene (444 bp), using a Gene‐Amp PCR System 9700 (Applied Biosystems, Forster City, CA). PCR reaction mixes and cycles are provided in Appendix S3. For each sample, 3 PCR reactions were performed with different DNA amounts to maximize the chances of having good amplifications. Amplification products of the 3 PCRs were pooled for the following steps. We performed nested PCRs when amplifications did not yield enough material. A first PCR round was performed with primers E8F (5′‐AGAGTTTGATCATGGCTCAG‐3′) and 907R (5′‐CCGTCAATTCTTTGAGTTT‐3′) amplifying a 900 bp fragment. Again, for each sample, 3 PCR reactions were performed with different DNA amounts to maximize the chances of amplifications (Appendix S3). A second nested PCR was performed on the amplicons of each of the 3 previous PCR using Whoi341 and Whoi785 (Appendix S3). The amplicons of the three nested PCR were pooled and purified with the Nucleospin Extract II (Macherey‐Nagel) following the instructions of the manufacturer. DNA was eluted in 60 μL (total) NE Buffer and stored at −20°C. Some negative PCR controls were also purified to be sequenced (Appendix S3). Amplification products were sent to the GeT PlaGe platform (Castanet‐Tolosan, France) for prokaryotic diversity sequencing using an Illumina MiSeq platform with a paired‐end read length of 2 × 250 bp with chemistry V3.

### 2.5 Metabarcoding Analyses

The DNA metabarcoding data were processed using SAMBA v3.0.1 (Standardized and Automated Metabarcoding Analyses [https://gitlab.ifremer.fr/bioinfo/workflows/samba]) an open‐source workflow using NextFlow workflow manager (Di Tommaso et al. 2017), which automates and standardizes the analysis of metabarcoding data through a suite of “standard” sequence treatment software. SAMBA is built around three main steps: data integrity checking, bioinformatics processes and statistical analyses. First, it consists in a checking process that allows to verify raw data integrity. Next, sequencing primers are trimmed from reads, and reads where primers are not found are removed, using QIIME 2 version 2019.10 (Bolyen et al. 2019). Then DADA2 (Callahan et al. 2016) is used to filter bad quality reads, correct sequencing errors, overlap paired reads, cluster sequences into ASV (Amplicon Sequence Variants) and remove chimeras. Here DADA2 was executed with the optimal paremeters. The dbOTU3 (Olesen, Duvallet, and Alm 2017) algorithm was used through QIIME 2. It clusters ASV according to sequence similarity and abundance profile in order to take into account the overestimation of diversity produced by DADA2. The *microDecon* R package (McKnight et al. 2019) is also used to remove contaminating ASVs present in control samples (extraction, PCR, purification blank). Taxonomic classification was achieved using the SILVA 138 reference database (Quast et al. 2012; Glöckner et al. 2017). Dominant ASVs were defined as the five most abundant ASVs within a bacterial genus for each symbiotic organ.

The final step of SAMBA consists in statistical analysis of α diversity and β diversity. We used observed richness (ASVs numbers) and Chao1 index (estimate the expected richness) to estimate the species richness within each sample based on ASVs dataset. ANOVA (Analysis of the Variance) tests were performed to compare the α diversity between species, sites and stages with both indexes. Only results with Chao1 are presented here since both gave identical results. β diversity analyses were achieved by ordination method using Non‐metric Multidimensional Scaling (NMDS) based on Jaccard distances (reflects bacterial communities composition) calculated on a normalized ASVs dataset (DESeq2 using standardization based on negative binomial distribution). Permutational multivariate analyses of variance (PERMANOVA) were performed using the Adonis2 function of package Vegan to assess the influence of site, species and life stage on bacterial community. Outside the SAMBA workflow, the tool ANCOM‐BC v2.0.3 (ANalysis of COmposition of Microbiomes with Bias Correction) is used to detect differentially abundant taxa at the genus and family level in different samples (Lin and Peddada 2020). The values obtained with ANCOM‐BC2 are characterized by “W” (W‐statistic, the test statistic of ANCOM‐BC2) that represents the standard error of the mean difference of absolute abundance between groups in log scale, *p* that represents the *p*‐value obtained from two‐sided *Z*‐test using the test statistic *W* and *q* that represents the adjusted *p*‐value. The R software (version 4.3.2) was used to perform statistical analyses and produce data visualizations.

BLASTN in Blast+ v2.12.0 (Camacho et al. 2009) was used to control the dominant ASVs affiliation to *Candidatus*_Hepatoplasma spp., *Deferribacteraceae* spp. and *Mariprofundus* spp. against the 16S rRNA sequences retrieved from MAGs obtained by Aubé et al. (2022) and Cambon‐Bonavita et al. (2021) using a num_alignments of 1.

### 2.6 Fluorescent In Situ Hybridization

Fluorescent in situ hybridization analyses were performed on dissected branchiostegites, scaphognathites, exopodites, midgut tube and foregut of juveniles at different stages (Appendices S1 and S4). Entire juveniles were also used. Fixed tissues were progressively dehydrated in PBS/ethanol series at ambient temperature, then transferred to ethanol/resin series at 40°C (Duperron et al. 2007). Dissected organs or the entire juveniles were then embedded in polyethylene glycol‐distearate—1‐hexadecanol (9:1) resin (Sigma‐Aldrich, Merck KGaA, Darmstadt, Germany). After polymerization, resin blocks containing the samples were stored at −20°C. They were trimmed into 8‐10 μm transversal tissue sections with a RM 2255 microtome (Leica Biosystems, Nussloch, Germany). Sections (between 3 and 7) were placed on slides (Menzel‐Gläser Superfrost Plus, USA). Before hybridization, resin was removed with ethanol (3 times 5 min in 96% ethanol) and tissues were partially rehydrated (5 min in 70% ethanol). Then tissues were hybridized in a reaction mix containing one or several FISH specific probes labeled with cyanine 3 (Cy3) or cyanine 5 (Cy5) dyes (Appendix S5) (at 0.5 μM final concentration) and the hybridization buffer [0.9 M NaCl, 0.02 M Tris–HCl [pH 7.5], 0.01% [w/v] sodium dodecyl sulphate (SDS), 20%, 30%, 35% or 45% deionized formamide], and incubated for 3 h at 46°C. After hybridization, sections were briefly pre‐rinsed in a washing buffer at 48°C, and washed in a rotary oven in the washing buffer at 48°C during 30 min. The washing buffer is adapted to the stringency condition used for hybridizations [0.215 M, 0.102 M, 0.07 M, 0.03 M NaCl respectively for 20%, 30%, 35% or 45% formamide, 0.02 M Tris–HCl [pH 7.5], 0.005 M EDTA [pH 8] and 0.01% [w/v] SDS]. After washing, the slides were briefly rinsed twice with distilled water: first at 48°C then at ambient temperature. Finally, sections were mounted with SlowFade Gold antifade reagent with DAPI (Invitrogen) and stored at −20°C in the dark. Observations of hybridized tissues were made using a Zeiss Axio Imager.Z2 microscope equipped with the Apotome.2 sliding module and Colibri.7 light technology (Zeiss, Oberkochen, Germany). Images were produced using the Zen software (Zeiss).

### 2.7 Scanning Electron Microscopy

Dissected foreguts and midgut tubes of *Rimicaris exoculata* and *Rimicaris chacei* stage A, B and subadult from TAG were used for electron microscopy. Glutaraldehyde fixed samples were dehydrated in ethanol series (10%–100% in 8 steps), before being placed in a filter holder (superposition of stainless‐steel washer and 0.2 μm polycarbonate filter) and critical‐point dried (Leica EM CPD300). Once dried, they were affixed to a stub using carbon glue and then coated by gold puttering (60% gold/40% Palladium, Quorum Technologies SC7640). SEM observations were performed using a Quanta 200 MK microscope (FEI, Hillsboro, OR) and images were taken with the Xt Microscope Control acquisition program (Soft Imaging System, Munster, Germany).

## 3 Results

### 3.1 PCR Comparison

The use of two distinct PCR approaches may bias our results (Kanagawa 2003). Therefore, a set of samples was amplified with both direct and nested PCRs in order to evaluate potential bias. According to the analysis, no significant bias was observed (Appendix S6, Figure S1, Tables S1–S3). This suggests that the PCR procedure did not significantly change the bacterial DNA amplification obtained, and samples amplified with both PCR procedures were further analyzed together.

### 3.2 Global Diversity of Symbiotic Tissues in Juveniles of Rimicaris Spp.

**FIGURE 1:**
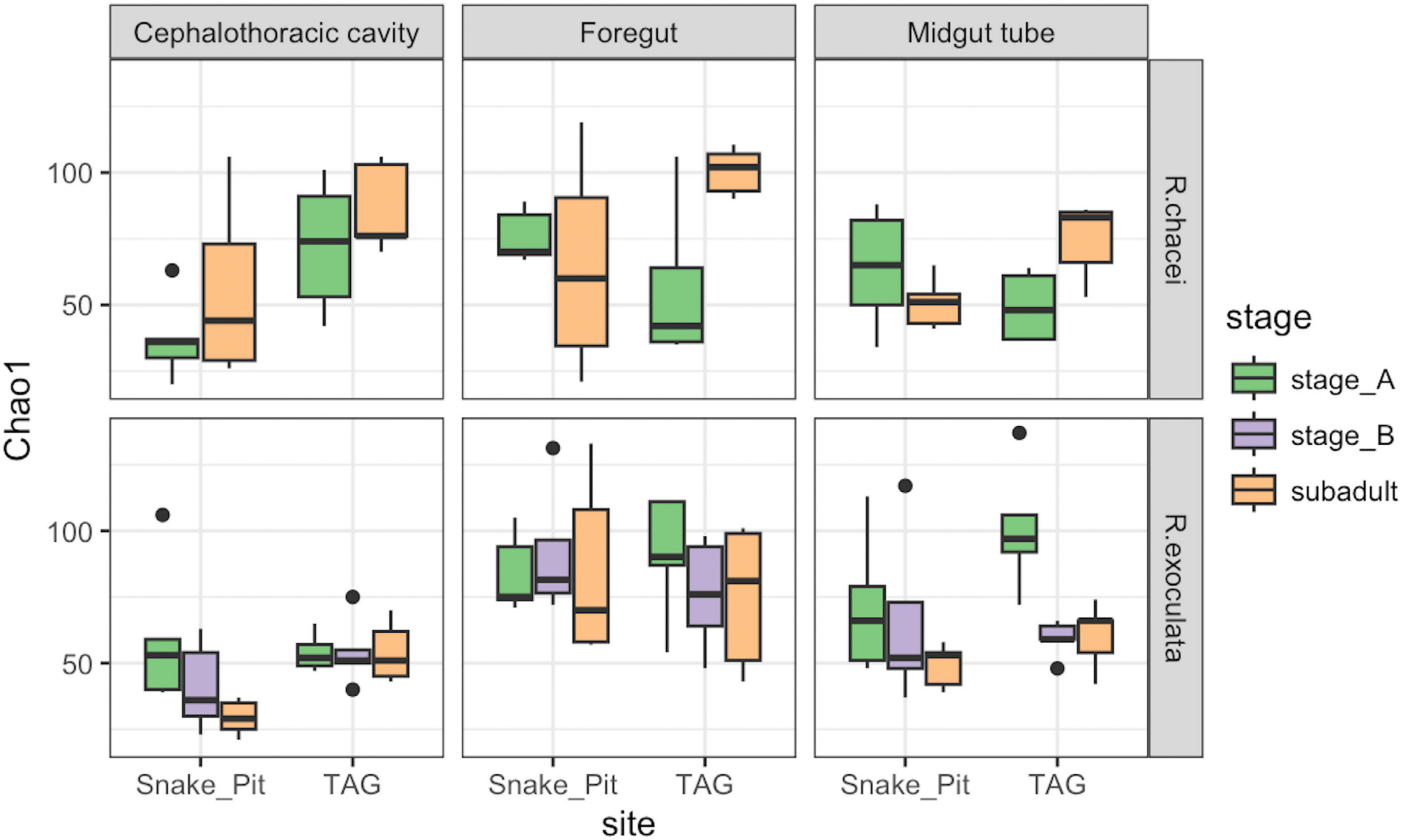
Boxplot for Chao1 richness estimator according to the species, the tissue, the stage, and the site.

First, all samples were considered together (Tables S4 and S5). Richness diversity analyses showed significant influence of the tissue (*p* = 2.23e‐06 with Chao1, ANOVA) and a slightest influence of the site (*p* = 0.0209 with Chao1, ANOVA) (Figure 1, Table S5). Moreover, to support these data, we realized a Tukey test. According to this test, significative difference were observed with the foregut and other tissues (Table S5). β diversity analyses showed that the diversity of symbiotic communities varied significantly with all considered factors: site, tissue, host species and life stages (*p* = 0.0001 each with Jaccard, Adonis2) (Table S5).

To study in more detail how symbiotic communities evolved along the development of each species, we analyzed metabarcoding data of each major symbiotic tissue separately: cephalothoracic cavity, foregut and midgut tube. Indeed, according to the diversity of the symbiotic communities observed in adults, and the results obtained with Richness and β diversities, each organ/cavity can be considered a proper functional entity within which we want to analyze the variations. In the following sections, an ASV table has then been produced for each tissue separately.

### 3.3 Digestive Anatomy and Symbiotic Communities of the Foregut

**FIGURE 2:**
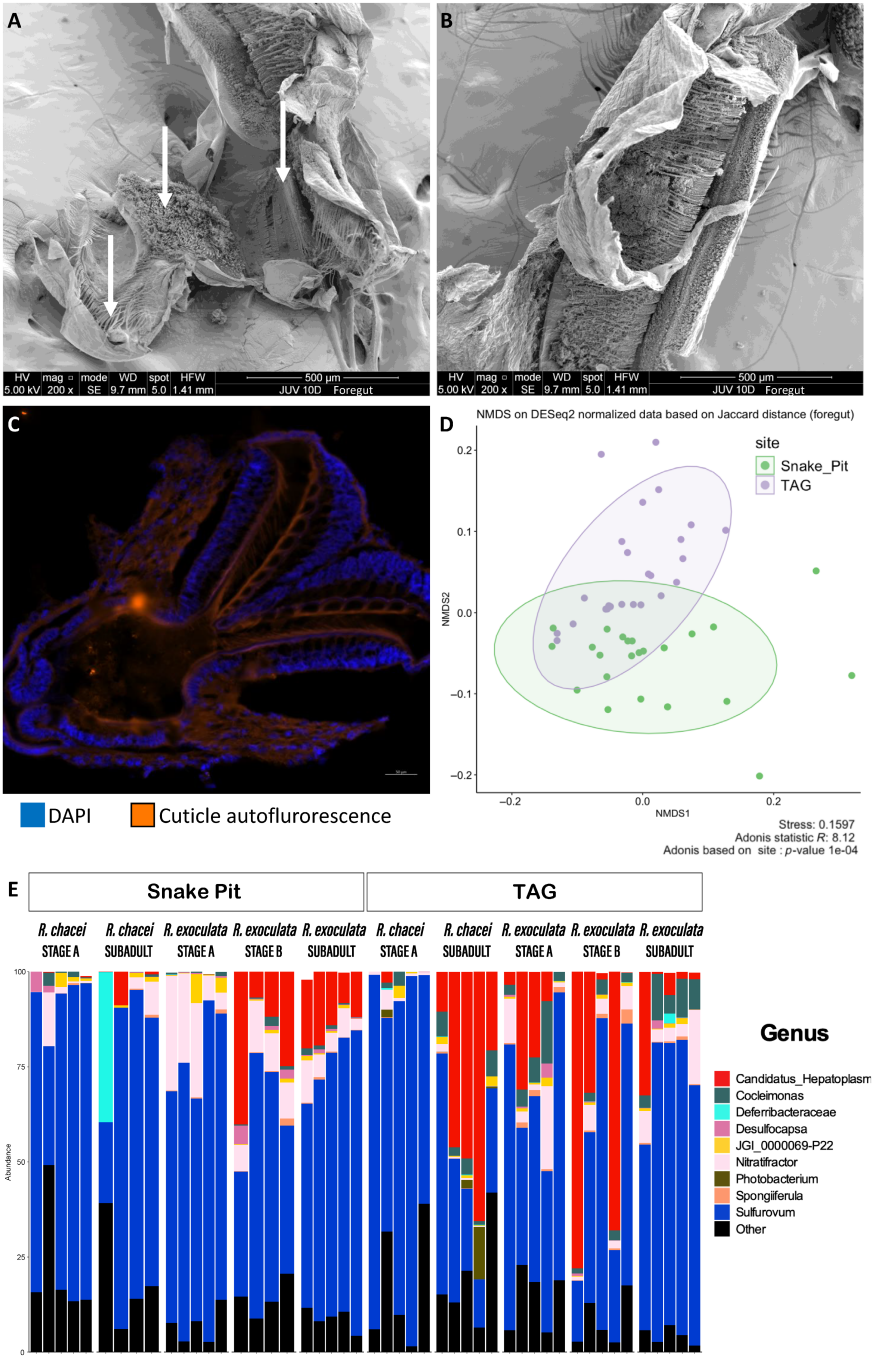
The foregut of *R. exoculata* and *R. chacei* juveniles. (A) Dorsal view of the pyloric chamber (opened) of a *R. exoculata* subadult with the different filtering structures showed by white arrows (SEM). (B) Dorsal view of the cardiac floor crest of the cardiac chamber of *R. exoculata* subadult specimen (SEM). (C) Section of the pyloric chamber of a *R. exoculata* stage A observed by autofluorescence at 555 nm thanks to the Filter Set 43 HE Cy3 (orange). Tissue cell nuclei are labeled with DAPI (blue). Scale bars = 50 μm (FISH). (D) NMDS plot of the bacterial diversity in foregut samples based on Jaccard distance at the ASV level and colored according to the site. Ellipses represent 95% confidence interval for each group. (E) Bacterial taxonomic composition at the genus‐level in foregut samples of juvenile shrimp at different life stages.

Stereomicroscope observations of whole juveniles stomach of each species show they look similar and only differ in size, those of *R. chacei* being slightly larger than those of *R. exoculata* at similar stage. The esophagus, as for adults, is really short, curved and narrow. This structure links the anterior part of the stomach to the shrimp's mouth. The stomach becomes larger in older stages, being in subadults roughly twice the size it is in stage A juveniles (Methou et al. 2023b). In all juvenile stages, the stomach forms a single cavity with two distinct parts, as for adults (Figure 2A). First, a large and smooth structure, the cardiac chamber (Figure 2B, Figure S2A,B), represents the larger part of the stomach (larger for *R. chacei* subadult specimens than *R*. exoculata subadult specimens). Between the cardiac chamber and the midgut tube, the pyloric chamber appears externally as a pair of striped “balls” (Figure 2A,C, Figure S2A). As for adults, the cardiac chamber is a simple structure covered of thin setae, well developed in stage A juveniles for both species. It is composed of a cardiac floor that is a complex filtration structure made of a superposition of ossicles and many setae (for complete description see Guéganton et al. 2022) (Figure 2B, Figure S2C). The pyloric chamber is also well developed in stage A juveniles for both species. It is composed of different complexe and filtering structures as the pylorus (pyramidal structure, Figure 2A, Figure S2D). This pyramid lies on the floor of the pyloric chamber and is made of different layers of plates, each covered with dense and serrulate setae (Figure 2A, Figure S2D). This filter is not attached to the cardiac floor. Rather, it is located under the floor, suggesting that the minerals and nutrient may fall along the cardiac floor crest directly into this pyramidal filter. On the sides of the pyramidal filter—the inner side of the lateral walls of the striped “balls”—there is a dense mat of setae, which are of two types (Figure 2A, Figure S2E,F). The most abundant look like the setae of the unpaired anterior ossicle—in the center of the mat—and the others are thinner and composed of several branches—on the periphery of the mat. There are minerals at the base of these mats (Figure S2E). On the ceiling of the pyloric chamber, other setae—very long, simple and thin—are visible on the cuticle and hang over the pyramidal filter (Figure S2F).

Our metabarcoding analyses showed that species richness of the foregut communities did not significantly vary with any of the considered factors (site, stage, species, respectively *p* = 0.933, *p* = 0.800 and *p* = 0.416, Choa1 index, ANOVA) (Table S6). Still, β diversity with NMDS showed a clear separation between vent sites (Figure 2D). In addition, it highlighted changes in foregut global bacterial diversity with life stage and a separation between species that was clear for older juveniles (stage B and subadult) while bacterial diversities in stage A were more similar and partially overlapped between the two species (Figure S3A,B). PERMANOVA analyses confirmed these observations. The bacterial community associated with the foregut was significantly influenced by the site (*p* = 0.0001, Jaccard index, Adonis2), the species (*p* = 0.0001, Jaccard index, Adonis2), and the life stage (*p* = 0.0012, Jaccard index, Adonis2) (Table S6).

Vizualization with barplots (Figure 2E) showed that bacterial taxonomic composition in the foregut seemed to be dominated by *Sulfurovum* spp. (Campylobacterales order, *Campylobacteria* class) in juveniles (85 ASVs out of 860), whatever the species and the life stage. Thirty‐one out these 85 ASVs are shared with those of the cephalothoracic cavity (ASV263, ASV336 and ASV1071, for example, in Table S4). *Candidatus* Hepatoplasma spp. were also one of the most represented lineages. It represented 25 ASVs out of the 860 detected. We compared the four dominant ASVs affiliated to *Candidatus* Hepatoplasma spp. to the 16S rRNA sequences retrieved from MAGs obtained by Aubé et al. 2022. These MAGs were retrieved in the foregut of *R. exoculata* adults and have been affiliated to two new candidate species. Among them, three main ASVs retrieved in the present study (AVS398, ASV1066 and ASV1074 in Table S4) were affiliated to *Candidatus* Foregutplasma rimicarensis (with 99.77%–100% sequence identity) and one (ASV959 in Table S4) to *Candidatus* Bg2_rimicarensis (100% sequence identity) that belong to the *Hepatoplasmataceae* (Aubé et al. 2022) (Table 1). These four dominant *Candidatus* Foregutplasma rimicarensis and *Candidatus* Bg2_rimicarensis ASVs were shared by the specimens from both species and sites with variations in their relative abundance throughout the life cycle (Figure 2E, Table S4). Indeed, they were almost absent in stage A and subadults *R. chacei* at Snake Pit, but were detected in higher proportions in subadults at TAG, even constituting a dominant lineage in some individuals (Figure 2E, Table 1). In *R. exoculata*, they were almost not detected in stage A juveniles from Snake Pit, but were already abundant in most individual of the same stages at TAG. Similarly to *R. chacei*, their proportions increased in later juvenile stages, but with high variability among individuals. In *R. exoculata* subadults, these four *Candidatus* Foregutplasma rimicarensis and *Candidatus* Bg2_rimicarensiswere also detected in high proportion, similar to those found in stage B juveniles at Snake Pit, but not at TAG where subadults generally exhibited low proportions of this lineage (except one individual) (Table 1, Figure 2E, Table S4). This result was confirmed by ANCOM‐BC2 analysis (Table S7). *Candidatus* Hepatoplasma spp. was significantly more abundant in later juvenile stage (*W* = 28.66, *q* = 2.51936e‐04 and *W* = 21.49, *q* = 9.01925e‐03, ANCOM‐BC2, respectively ASV1074 and ASV959). Even if the metabarcoding analysis highlighted the presence of *Candidatus* Foregutplasma rimicarensis and *Candidatus* Bg2_rimicarensis in the foregut (Table 1), they were not visualized in FISH with Myco378‐1 in any juvenile stages of the two shrimp species (Figure 2C). Neither in the esophagus, nor in the stomach the probe gave any fluorescent signal. In some subadult individuals of *R. exoculata* from TAG, Myco378‐1 (Guéganton et al. 2022) highlighted some coccoid bacteria in the alimentary bolus or near the tissues. Still, the high mineral content in the alimentary bolus may hide the bacteria and prevent hybridization signal visualization due to autofluorescence. Fourteen ASVs out of 860 ASVs retrieved in the foregut were affiliated to *Deferribacteraceae* spp. Twelve of them were being shared with those colonizing the midgut tube. These 14 ASVs were mostly found in two subadults specimens only (a *R. chace*i from Snake Pit and a *R. exoculata* from TAG, ASV432, ASV685, ASV827 in Table S4, Figure 2E).

**TABLE 1:** Proportion of the main ASVs (3 corresponding to *Candidatus* Foregutplasma rimicarensis and 1 corresponding to *Candidatus* Bg2_rimicarensis) obtained and affiliated to the genus *Candidatus* Hepatoplasma of the total of bacterial communities of foregut samples in juveniles of the two *Rimicaris* spp. at different life stages (average ± standard deviation).

	*R. chacei* stage A	*R. chacei subadult*	*R. exoculata* stage A	*R. exoculata* stage B	*R. exoculata subadult*
TAG					
*Candidatus* Foregutplasma rimicarensis (%)	0.00 ± 0.00 (*n* = 5)	34.68 ± 24.92 (*n* = 5)	12.89 ± 13.24 (*n* = 5)	35.28 ± 36.15 (*n* = 5)	8.07 ± 13.36 (*n* = 5)
*Candidatus* Bg2_rimicarensis (%)	0.56 ± 1.25 (*n* = 5)	3.63 ± 4.83 (*n* = 5)	0.001 ± 0.003 (*n* = 5)	0.34 ± 0.46 (*n* = 5)	0.37 ± 0.52 (*n* = 5)
Snake Pit					
*Candidatus* Foregutplasma rimicarensis (%)	0.07 ± 0.10 (*n* = 5)	0.08 ± 0.14 (*n* = 3)	0.00 ± 0.00 (*n* = 5)	18.82 ± 15.63 (*n* = 4)	12.21 ± 4.88 (*n* = 5)
*Candidatus* Bg2_rimicarensis (%)	0.00 ± 0.00 (*n* = 5)	1.76 ± 3.30 (*n* = 4)	0.006 ± 0.01 (*n* = 5)	2.00 ± 2.88 (*n* = 4)	1.30 ± 2.35 (*n* = 5)

### 3.4 Digestive Anatomy and Symbiotic Communities of the Midgut Tube

**FIGURE 3:**
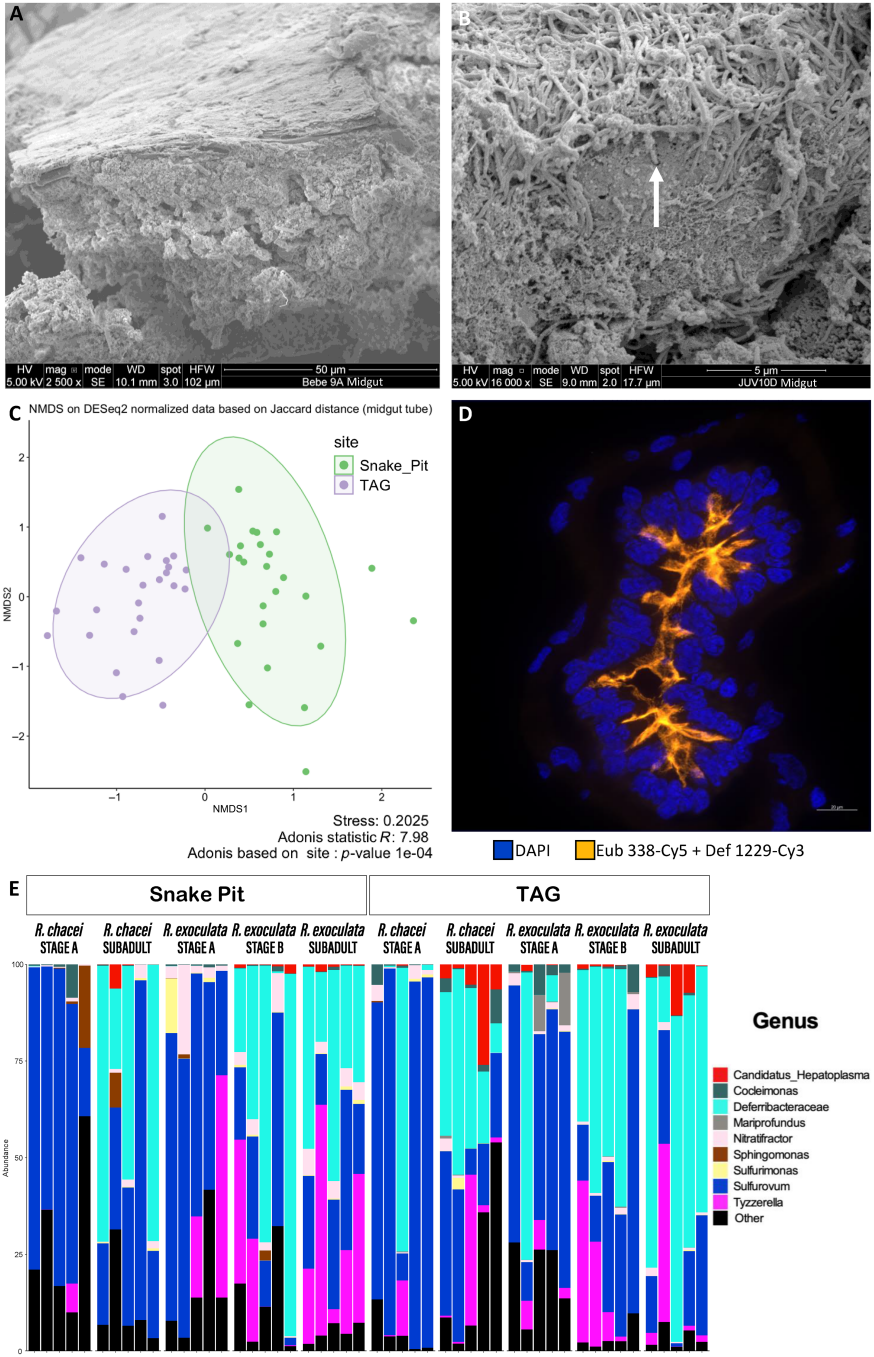
The midgut tube of *R. exoculata* and *R. chacei* juveniles. (A) General structure of a section of the midgut tube of a *R. chacei* stage A specimen. No microvilli is visible (SEM). (B) Zoom on a section of the epithelium of the midgut tube of a *R. exoculata* subadult specimen. The white arrow shows the insertion of bacteria between the microvilli (SEM). (C) NMDS plot of the bacterial diversity in midgut tube samples based on Jaccard distance at the ASV level and colored according to the site. Ellipses represent 95% confidence interval for each group. (D) *Candidatus* Microvillispirillaceae (light‐orange) of the midgut tube of a *R. exoculata* subadult from Snake Pit co‐hybridized with the specific probes Def1229‐Cy3and Eub338‐Cy5. The superposition of the probe gives orange colored bacteria. Tissue cell nuclei are labeled with DAPI (blue). Scale bars = 20 μm (FISH). (E) Bacterial taxonomic composition at the genus‐level in midgut samples of juvenile shrimp at different life stages.

The structure of midgut tubes from stage A juveniles of both species clearly differed from midgut tubes of other stages with no visible microvilli (Figure 3A). On the contrary, the midgut tube was well developed for stages B and subadults of *R. exoculata* and subadults of *R. chacei* with microvilli of the epithelial cells already visible (Figure 3B, Figure S3A–C). They form a thick and dense layer between the epithelium and the alimentary bolus that is full of minerals.

Our metabarcoding analyses showed no significant variation in bacterial richness with life stages, species, or site (respectively, *p* = 0.251, *p* = 0.113 and *p* = 0.163, Chao1 index, ANOVA) (Table S8). Similar to foregut communities, NMDS showed a clear separation at the ASV level of midgut community compositions between vent sites and also highlighted changes in life stages and separation between species (Figure 3C, Figure S3C,D). A PERMANOVA analysis confirmed that the site, the shrimp species (*p* = 0.0001 for both, Jaccard index, Adonis2), and the stage (*p* = 0.0002, Jaccard index, Adonis2) all significantly influenced bacterial communities' structure (Table S8).

As for the foregut, *Sulfurovum* spp. (*Campylobacte*rales order) were detected in all specimens with 69 ASVs out of 814, being in large proportion or even dominant in most samples (Figure 3E, Table S4). Forty‐one of these 69 ASVs are also shared with the ones of the foregut and 28 of these 41 are retrieved also in the cephalothoracic cavity (ASV263, ASV336 and ASV1071, for example, in Table S4). Two other lineages were also well represented: *Tyzzerella* spp. with 11 ASVs out of 814 (*Clostridia* class) and *Deferribacteraceae* spp. with 21 ASVs out of 814 (*Deferribacteres* class) (Table S4). We compared the main *Deferribacteraceae* spp. ASVs against the 16S rRNA sequences retrieved from symbiont MAGs obtained by Aubé et al. (2022). These MAGs were retrieved in the midgut of *R. exoculata* adults. We identified that the 5 main ASVs (ASV432, ASV685, ASV827, ASV992 and ASV1214 in Table S4) were affiliated to the genus *Candidatus* Rimicarispirillum spp. (with 99.5%–100% sequence identity) and 2 other ASVs (ASV45 and ASV251 in Table S4) were 96% identical to 16S sequences within the MAGs obtained by Aubé et al. (2022). These two ASVs belong to the *Candidatus* Microvillispirillaceae family. In all samples, most *Deferribacteraceae* spp. were affiliated to the five dominant *Candidatus* Rimicarispirillum spp. Other *Candidatus* Microvillispirillaceae lineages were almost not found at TAG, but occurred in significant proportions at Snake Pit, although *Candidatus* Rimicarispirillum remained the dominant *Deferribacteraceae* spp. at both sites (Table 2, Figure 3E). *Candidatus* Microvillispirillaceae were almost not detected in stage A juveniles except in one out of 10 individuals of each species at TAG where they dominated midgut communities (Table 2, Table S4). These data were supported by SEM: no bacteria was visible whatever species or site, as well as by FISH (probe Eub338 and specific probe Def1229, Amann et al. 1990; Guéganton et al. 2022) with no *Candidatus* Microvillispirillaceae observed in any of the midgut tube sections of stage A juveniles (Figure S4D). In contrast, *Candidatus* Microvillispirillaceae and more precisely *Candidatus* Rimicarispirillum spp. were found in large proportions in almost all later juvenile stages and subadults at both sites (ANCOM‐BC2, *W* = 34.63, *q* = 1.06297e‐05 and *W* = 23.92, *q* = 2.22081e‐03, respectively, ASV432 and ASV1214 in Table S9, Table 2). According to SEM observations, long and well‐developed bacteria with the typical *Candidatus* Microvillispirillaceae morphology attached to the tissue, extending out and entangled within the microvilli, were visible in both stages B and subadults (Figure 3B, Figure S4A–C). The same bacteria, appearing as long “spaghetti‐like” cells, were observed in FISH with the specific probe Def1229 in stage B and subadut for *R. exoculata* and *R. chacei* (Snake Pit and TAG) (Figure 3D, Figure S4E,F). They were not visible all along the midgut tube, but were mostly observed in the posterior part of the midgut tube close to the hindgut, whereas the anterior part seemed uncolonized yet. They were separated from the alimentary bolus by the peritrophic membrane as observed for adults (Durand et al. 2009; Guéganton et al. 2022). *Tyzzerella* spp. were detected in varying proportions among individuals and the most dominant ASV (ASV110 in Table S4) tended to be more present in *R. exoculata* and shared by almost all specimens from TAG especially. *Candidatus* Hepatoplasma spp. (8 ASVs out of 814) were identified in the midgut of some specimens (mainly subadults at TAG) (Figure 3E, Table S9). Among these 8 ASVs, only four with two dominants were shared with the foregut for stage B and subadult specimens (ASV959 and ASV1074 in Table S4).

**TABLE 2:** Proportion of the main ASVs (5 corresponding to *Candidatus* Rimicarispirillum and 2 corresponding to *Candidatus* Microvillispirillaceae)and affiliated to the genus *Deferribacteraceae* of the total of bacterial communities of midgut tube samples in juveniles of the two *Rimicaris* spp. at different life stages (average ± standard deviation).

	*R. chacei* stage A	*R. chacei subadult*	*R. exoculata* stage A	*R. exoculata* stage B	*R. exoculata subadult*
TAG					
*Candidatus* Rimicarispirillum spp. (%)	13.82 ± 29.92 (*n* = 5)	26.62 ± 15.55 (*n* = 5)	14.92 ± 30.15 (*n* = 5)	39.85 ± 23.63 (*n* = 5)	54.34 ± 25.60 (*n* = 5)
Others dominant *Candidatus* Microvillispirillaceae ASVs (%)	0.00 ± 0.00 (*n* = 5)	0.00 ± 0.00 (*n* = 5)	0.001 ± 0.003 (*n* = 5)	0.00 ± 0.00 (*n* = 5)	0.00 ± 0.00 (*n* = 5)
Snake Pit					
*Candidatus* Rimicarispirillum spp. (%)	0.027 ± 0.06 (*n* = 5)	26.99 ± 25.25 (*n* = 5)	0.01 ± 0.02 (*n* = 5)	40.12 ± 35.43 (*n* = 5)	29.54 ± 9.66 (*n* = 5)
Others dominant *Candidatus* Microvillispirillaceae ASVs (%)	0.00 ± 0.00 (*n* = 5)	16.29 ± 19.32 (*n* = 5)	0.00 ± 0.00 (*n* = 5)	5.30 ± 8.08 (*n* = 5)	5.71 ± 9.43 (*n* = 5)

### 3.5 Symbiotic Communities of the Cephalothoracic Cavity

**FIGURE 4:**
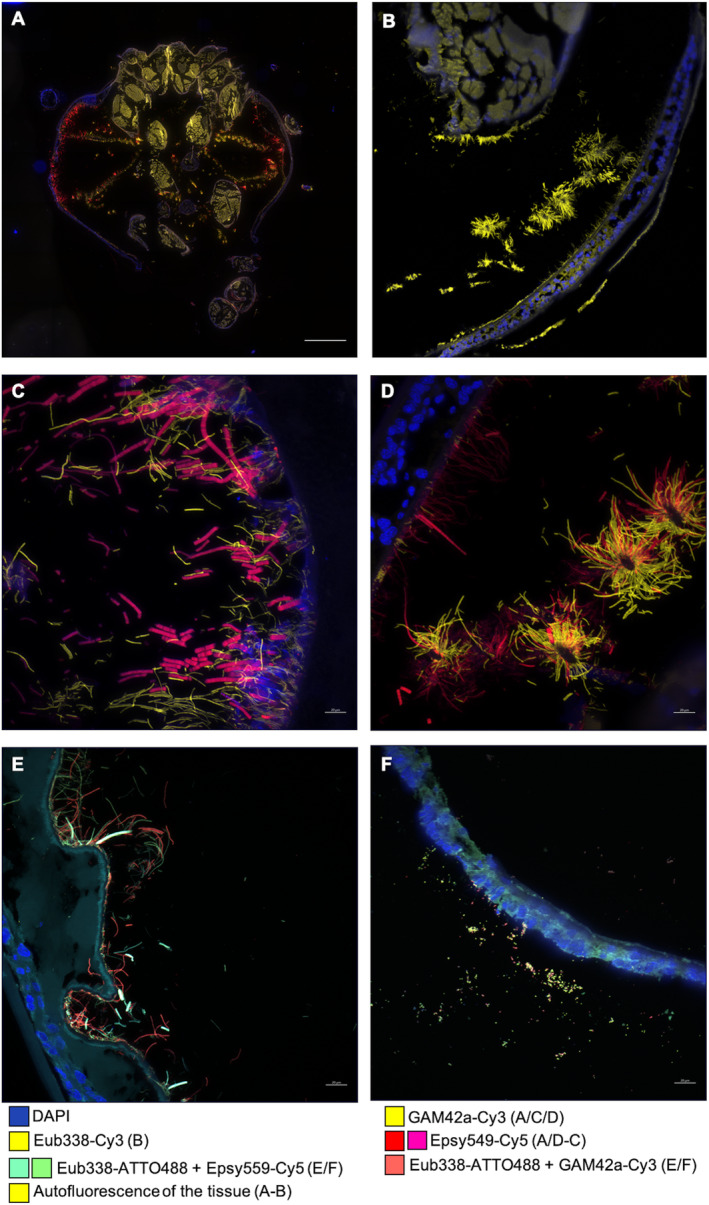
FISH observations of cephalothoracic symbionts evolution for both species during metamorphosis. *Gammaproteobacteria* were hybridized with the specific probe GAM42a and *Campylobacteria* with Epsy549. (A) Entire cephalothoracic cavity of a *R. exoculata* from TAG hybridized with the specific probes GAM42a‐Cy3 (yellow) and Epsy549‐Cy5 (red). Autofluorescence of the tissus is also observable in yellow. (B) Entire cephalothoracic cavity of a *R. chacei* from Snake Pit hybridized with the general probe Eub338‐Cy3 (yellow). Autofluorescence of the tissus is also observable in yellow. (C) Bacteria on branchiostegites of a *R. exoculata* subadult from TAG hybridized with the specific probes Epsy549‐Cy5 (pink) and GAM42a‐Cy3 (yellow). (D) Bacteria on branchiostegites and setae of the scaphognathite of a *R. chacei* subadult from TAG hybridized with the specific probes Epsy549‐Cy5 (red) and GAM42a‐Cy3 (yellow). (E) Bacteria on branchiostegites of a *R. exoculata* stage A from TAG co‐hybridized with the specific probes Epsy549‐Cy5/Eub338‐ATTO488 (green‐blue) and GAM42a‐Cy3/Eub338‐ATTO488 (red). (F) Bacteria on branchiostegites of a *R. chacei* stage A from Snake Pit co‐hybridized with the specific probes Epsy549‐Cy5/Eub338‐ATTO488 (green‐blue) and GAM42a‐Cy3/Eub338‐ATTO488 (red). Tissue cell nuclei were labeled with DAPI (blue). (A, B) were mosaics. Scale bars = 20 μm (C–F), 500 μm (A, B).

In both species, we observed increasing bacterial colonization of the cephalothoracic cavity along the host post‐settlement development (Figure 4). Indeed, the mouthparts and the branchiostegites were generally colonized by much denser bacterial communities in subadults than in stage A juveniles. In addition, filamentous bacteria presented more subunits and were more abundant in subadults compared to juvenile stages (Figure 4). Overall, *R. chacei* were less colonized than *R. exoculata* at equivalent life stages. Cephalothoracic cavities of *R. chacei* stage A juveniles were mostly uncolonized whereas dense bacterial coverage was observed in *R. exoculata* of a similar stage (Figure 4E,F). Mouthparts (scaphognathites and exopodites) and their setae were fully colonized in subadults of both species (Figure 4A–D). However, only branchiostegites of *R. exoculata* subadults were fully colonized, whereas those of *R. chacei* exhibited limited bacterial colonization (Figure 4A–D).

**FIGURE 5:**
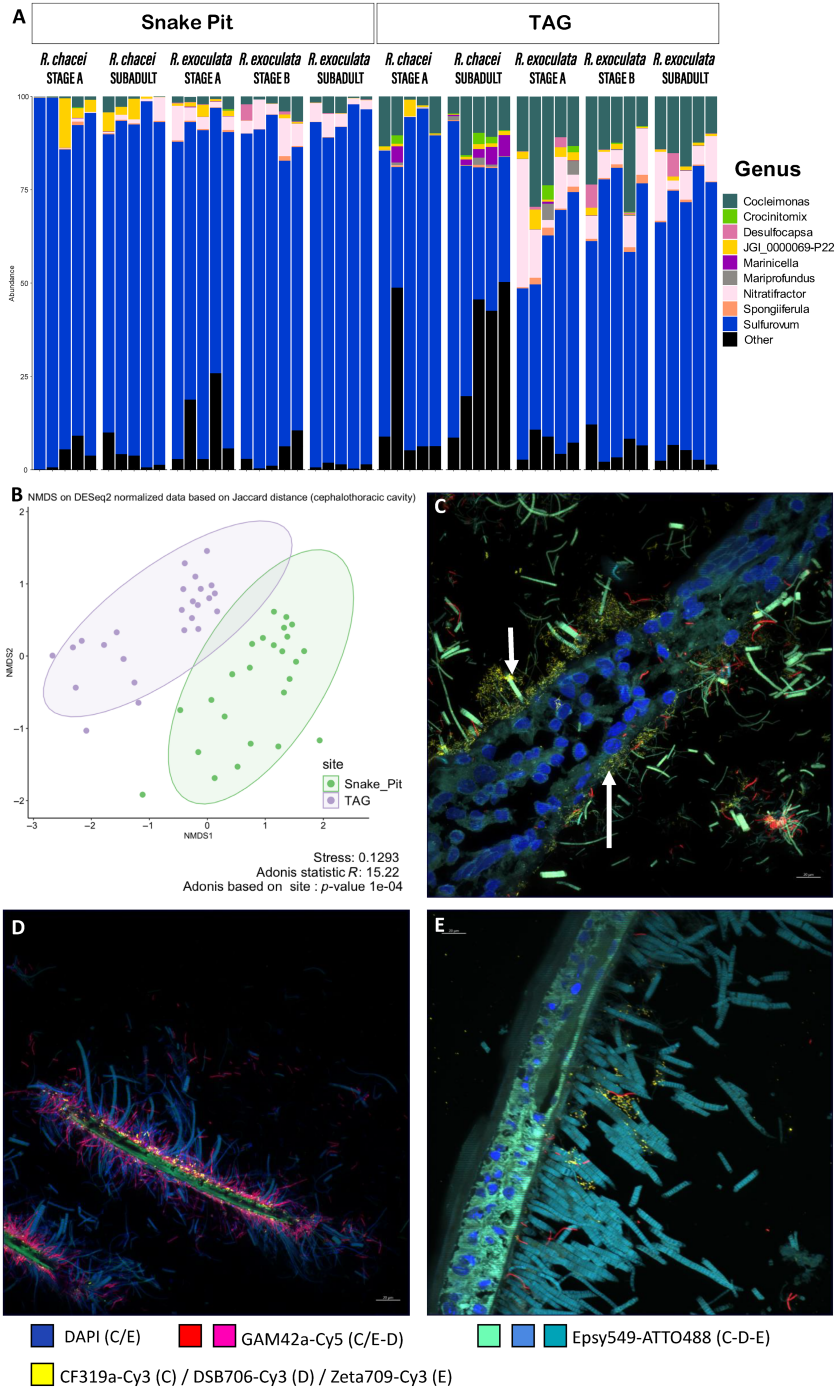
The cephalothoracic cavity of *R. exoculata* and *R. chacei* juveniles. Using FISH, *Gammaproteobacteria* were hybridized with the specific probe GAM42a, *Campylobacteria* with Epsy549, *Zetaproteobacteria* with Zeta709, *Bacteroidia* with CF319a and *Desulfobulbia* with DSB706. (A) Bacterial taxonomic composition at the genus‐level in cephalothorax samples of juvenile shrimp at different life stages. (B) NMDS plot of the bacterial diversity in cephalothorax samples based on Jaccard distances at the ASV level and colored according to the site. Ellipses represent 95% confidence interval for each group. (C) Bacteria on scaphognathites of a *R. exoculata* stage B from Snake Pit hybridized with the specific probes Epsy549‐ATTO488 (green), GAM42a‐Cy5 (red) and CF319a‐Cy3 (yellow, white arrows). (D) Bacteria on setae of scaphognathites of a *R. exoculata* stage B from Snake Pit hybridized with the specific probes Epsy549‐ATTO488 (blue), GAM42a‐Cy5 (red) and DSB706‐Cy3 (yellow). (E) Bacteria on branchiostegites of a *R. exoculata* stage B from Snake Pit hybridized with the specific probes Epsy549‐ATTO488 (green‐blue), GAM42a‐Cy5 (red) and Zeta709‐Cy3 (yellow). Tissue cell nuclei were labeled with DAPI (blue). Scale bars = 20 μm.

Richness diversity analyses showed that the site and species factors (respectively, *p* = 0.003454 and *p* = 0.04336, Chao1 index, ANOVA) significantly impact bacterial richness in the cephalothoracic cavity (Table S10). NMDS plots showed a clear separation at the ASV level between vent sites and between species, but changes among life stages were less marked and overlapped largely within each *Rimicaris* species (Figure 5B, Figure S3D,E). Accordingly, β diversity analyses showed that site, species (*p* = 0.0001 for both, Jaccard index, Adonis2), and life stage (*p* = 0.0034 each, Jaccard index, Adonis2) all had a significant influence on the bacterial community composition in the cephalothorax (Table S10).

Taxonomic composition of bacterial communities from the cephalothoracic cavity in all juveniles was similar to those found in adults (Figure 5A). *Nitratifractor* spp. (13 ASVs out of 515) and *Sulfurovum* spp. (54 ASVs out of 515) (Campylobacterales order, *Campylobacteria* class) were retrieved whatever the site, the stage and the species (except for some stage A and subadult specimens of *R. chacei*). The main lineage was *Sulfurovum* spp. for both host species. Dominant ASVs (ASV390 and ASV483, for example, in Table S4) were shared by almost all the specimens whereas other (such as ASV82 and ASV824 in Table S4) rather colonized specimens from Snake Pit whatever the stage (Table 3). *R. exoculata* specimens showed more *Nitratifractor* spp. related sequences in their microbial communities than *R. chacei* (ANCOM‐BC, *W* = 6.21, *q* = 7.40199e‐08 and *W* = 9.86, *q* = 9.46443–21, respectively ASV994 and ASV527 in Table S11, Table 3) shared throughout life cycle (as ASV527 or ASV994 in Table S4). Using FISH (specific probe Epsy549, Lin et al. 2006), as for adults, *Sulfurovum* spp. were mostly observed on the inner side of the branchiostegites or on setae (filament composed of multiple cell units, Figure 4A–C, Figure S5). *Cocleimonas* spp. (34 ASVs out of 515) and *Marinicella* spp. (6 ASVs out of 515) (*Gammaproteobacteria* class) were retrieved whatever the site, life stage or species (Table 3, Tables S4). ASVs affiliated to *Cocleimonas* spp. were significantly more retrieved at TAG (ANCOM‐BC2, ASV562, for example, *W* = 11.49, *q* = 2.30758e‐28, in Table S12, Table 3). The same dominant ASVs (ASV193, ASV916 or ASV1023 Table S4) were found all along the different life stages at TAG. Using FISH (specific probe GAM42a, Manz et al. 1992) at subadult stage for both species, they were observable (thin filaments, sometimes bacillis or coccoids) mainly on the setae of scaphognathites and exopodites (low abundancy) whereas branchiostegites were almost deprived of these lineages at Snake Pit. On the contrary at TAG, they were found in higher abundancy, mainly on the setae and on the branchiostegites (Figure 4, Figure S5). *Crocinitomix* spp. (5 ASVs out of 515) and *Spongiiferula* spp. (5 ASVs out of 515) (*Bacteroidia* class) were retrieved whatever the site, the species and the stage (except for *R. exoculata* subadult specimens from Snake Pit for *Crocinitomix* spp., Table 3, Table S4), even if they were mostly retrieved at TAG compared to Snake Pit (ANCOM‐BC2, ASV466, *W* = −3.89, *q* = 1.14225e‐02 for *Crocinitomix* spp., ASV732, *W* = 9.21, *q* = 4.84954e‐18 and ASV113, *W* = −3.52, *q* = 4.75675e‐02 for *Songiiferula* spp., in Table S12). The results were confirmed using FISH (specific probe CF319a, Manz et al. 1996). Most of the time, these bacteria were located on the scaphognathites, exopodites and their setae, or on the inner side of the branchiostegites. Moreover, they were always close to *Sulfurovum* spp. (Figure 5 C, Figure S5A,B), often at the base of the tissues or of the setae *Desulfocapsa* spp. (4 ASVs out of 515) (*Desulfobulbales* order, *Desulfobulbia* class) were identified in *R. exoculata* at each stage and site (except for one subadult from Snake Pit and one stage B and one stage A from TAG), and for *R. chacei* in 2 stage A juveniles Snake Pit respectively, and in a subadult from Snake Pit (Table 3, Table S4). For some specimens, especially for *R. chacei*, *Desulfocapsa* spp. ASVs account for only a few sequences (Table 3). Using FISH (specific probe DSB706, Lucker et al. 2007), they could be only found in stage B juveniles at Snake Pit and subadults at both sites for *R. exoculata* in the form of little coccoids (Figure 5D, Figure S5C). They were mixed with the *Sulfurovum* spp. and *Cocleimonas* spp. at the bottom of the microbial mat, and more particularly at the base of the filamentous bacteria both on branchiostegites and all around the setae of the scaphognathites and exopodites (Figure 5D, Figure S5C). *Mariprofondus* spp. (3 ASVs out of 515) (*Zetaproteobacteria* class) were detected mainly at TAG (but slightly in some juveniles from Snake Pit) (Table 3, Table S4). At TAG, these were identified on all specimens of every stages of *R. chacei* but only in some for *R. exoculata* (all stage A, 4/5 stage B and 2/5 subadults) (Table S4). As for the foregut and the midgut tube, we compared the three *Mariprofondus* spp. (ASV638, ASV799 and ASV1255 in Table S4) ASVs against the 16S rRNA sequences retrieved from symbiont MAGs obtained by Cambon‐Bonavita et al. 2021. These MAGs were retrieved in the cephalothoracic cavity of adults. We identified that the ASVs were affiliated to the genus *Candidatus* Ghiorsea crypta (with 98.126%–100% sequence identity). Using FISH (specific probe Zeta709, Hoshino et al. 2016), they were visible in stage B juveniles and subadults of *R. exoculata* and in *R. chacei* subadults at both sites (less visible at Snake Pit) but could not be observed in stage A juveniles of both species. They were rod‐shaped bacteria, found in low numbers along the setae and tissues of scaphognathites and exopodites as well as on the inner side of the branchiostegites (Figure 5E, Figure S5D). Table S4 showed that only three ASVs of *Candidatus*_hepatoplasma spp. from the foregut (ASV287, ASV959 and ASV1074) and five AVSs of *Deferribacteraceae* spp. (ASV251, ASV432, ASV685, ASV827 and ASV1214) from the midgut were retrieved in the cephalothoracic cavity in very small proportion only in few subalduts specimens, showing these bacaterial lineages are not shared between the cephalothoracic cavity and the digestive organs.

**TABLE 3:** Proportion of sequences obtained and affiliated to the main bacterial communities of the cephalothoracic cavity samples in juveniles of the two *Rimicaris* spp. at different life stages (average ± standard deviation).

	*R. chacei* stage A	*R. chacei subadult*	*R. exoculata* stage A	*R. exoculata stage* B	*R. exoculata subadult*
TAG					
*Clocleimonas* spp. (%)	7.31 ± 5.44 (*n* = 5)	9.94 ± 4.00 (*n* = 5)	18.39 ± 7.89 (*n* = 5)	17.78 ± 9.28 (*n* = 5)	14.02 ± 3.28 (*n* = 5)
*Crocinitomix* spp. (%)	0.49 ± 0.99 (*n* = 5)	1.11 ± 1.23 (*n* = 5)	1.12 ± 1.69 (*n* = 5)	0.14 ± 0.13 (*n* = 5)	0.02 ± 0.04 (*n* = 5)
*Desulfocapsa* spp. (%)	0.00 ± 0.00 (*n* = 5)	0.00 ± 0.00 (*n* = 5)	0.75 ± 1.15 (*n* = 5)	1.34 ± 2.72 (*n* = 5)	1.32 ± 2.78 (*n* = 5)
*JGI_0000069‐P22* spp. (%)	1.38 ± 1.74 (*n* = 5)	0.85 ± 0.43 (*n* = 5)	2.46 ± 1.76 (*n* = 5)	0.91 ± 0.79 (*n* = 5)	0.76 ± 0.20 (*n* = 5)
*Marinicella* spp. (%)	0.88 ± 1.91 (*n* = 5)	2.93 ± 2.25 (*n* = 5)	0.17 ± 0.23 (*n* = 5)	0.04 ± 0.03 (*n* = 5)	0.04 ± 0.03 (*n* = 5)
*Candidatus* Ghiorsea crypta (%)	0.23 ± 0.28 (*n* = 5)	0.74 ± 0.74 (*n* = 5)	1.63 ± 2.18 (*n* = 5)	0.13 ± 0.12 (*n* = 5)	0.03 ± 0.05 (*n* = 5)
*Nitratifractor* spp. (%)	0.11 ± 0.11 (*n* = 5)	0.02 ± 0.02 (*n* = 5)	13.26 ± 12.95 (*n* = 5)	7.56 ± 3.19 (*n* = 5)	9.16 ± 6.38 (*n* = 5)
*Spongiiferula* spp. (%)	0.12 ± 0.19 (*n* = 5)	0.31 ± 0.26 (*n* = 5)	1.21 ± 0.83 (*n* = 5)	1.09 ± 0.76 (*n* = 5)	0.40 ± 0.17 (*n* = 5)
*Sulfurovum* spp. (%)	74. 37 ± 24.13 (*n* = 5)	50.72 ± 22.18 (*n* = 5)	54.25 ± 12.19 (*n* = 5)	64.53 ± 13.92 (*n* = 5)	70.55 ± 6.33 (*n* = 5)
Snake Pit					
*Clocleimonas* spp. (%)	0.87 ± 1.17 (*n* = 5)	1.52 ± 1.84 (*n* = 5)	1.80 ± 1.03 (*n* = 5)	3.06 ± 2.32 (*n* = 5)	2.29 ± 1.99 (*n* = 5)
*Crocinitomix* spp. (%)	0.05 ± 0.10 (*n* = 5)	0.01 ± 0.03 (*n* = 5)	0.09 ± 0.15 (*n* = 5)	0.002 ± 0.004 (*n* = 5)	0.00 ± 0.00 (*n* = 5)
*Desulfocapsa* spp. (%)	0.009 ± 0.02 (*n* = 5)	0.01 ± 0.03 (*n* = 5)	0.10 ± 0.06 (*n* = 5)	1.07 ± 1.84 (*n* = 5)	0.09 ± 0.08 (*n* = 5)
*JGI_0000069‐P22* spp. (%)	3.87 ± 5.43 (*n* = 5)	2.68 ± 2.55 (*n* = 5)	1.40 ± 1.09 (*n* = 5)	0.30 ± 0 0.42 (*n* = 5)	0.08 ± 0.04 (*n* = 5)
*Marinicella* spp. (%)	0.03 ± 0.04 (*n* = 5)	0.02 ± 0.03 (*n* = 5)	0.07 ± 0.07 (*n* = 5)	0.006 ± 0.01 (*n* = 5)	0.002 ± 0.005 (*n* = 5)
*Candidatus* Ghiorsea crypta (%)	0.00 ± 0.00 (*n* = 5)	0.00 ± 0.00 (*n* = 5)	0.02 ± 0.02 (*n* = 5)	0.00 ± 0.00 (*n* = 5)	0.00 ± 0.00 (*n* = 5)
*Nitratifractor* spp. (%)	0.27 ± 0.35 (*n* = 5)	1.94 ± 2.49 (*n* = 5)	4.24 ± 2.94 (*n* = 5)	6.09 ± 3.10 (*n* = 5)	3.75 ± 1.88 (*n* = 5)
*Spongiiferula* spp. (%)	0.24 ± 0.38 (*n* = 5)	0.26 ± 0.12 (*n* = 5)	0.34 ± 0.15 (*n* = 5)	0.34 ± 0.49 (*n* = 5)	0.07 ± 0.06 (*n* = 5)
*Sulfurovum* spp. (%)	90.82 ± 8.88 (*n* = 5)	89.57 ± 6.58 (*n* = 5)	80.68 ± 7.46 (*n* = 5)	84.88 ± 8.29 (*n* = 5)	92.55 ± 4.07 (*n* = 5)

## 4 Discussion

Our study provides a first comprehensive dataset of symbiotic communities colonizing each symbiotic organ (midgut tube, foregut, cephalothoracic cavity) of the distinct juvenile stages of *Rimicaris chacei* and *Rimicaris exoculata*. While previous studies were limited in the number of specimens available as well as by the lack of a precise identification of the distinct juvenile stages (Guri et al. 2012; Cowart et al. 2017; Apremont et al. 2018), our dataset with 5 replicates per species/stage/site, using the revised identification of *Rimicaris* juveniles (Methou et al. 2020), allows us to investigate the inter‐individual variability and the influence of biotic (species, stage) and environmental (site) factors on symbiotic development in different host organs.

### 4.1 Anatomical Change of Digestive Organs Upon Symbiont Acquisition

Morphologically, our observations showed that the stomach structures of juveniles were entirely developed even if it was of smaller size than in adults (Guéganton et al. 2022). On the contrary, the midgut tube was devoid of microvilli in the earliest stage (stage A) of both species, appearing somewhat immature. These structures only appeared in older juveniles (respectively, stage B juveniles for *R. exoculata* and subadults for *R. chacei*). The formation of these microvilli was concomitant with the specific midgut symbionts colonization. Involvement of symbionts in metamorphosis and maturation of animal tissues has been observed in many species across nearly all metazoan phyla suggesting that symbionts act as “the other cells” of their host developmental machinery (Carrier and Bosch 2022). In the squid *Euprymna scolopes* symbiosis with the bioluminescent bacteria *Vibrio fischeri*, aposymbiotic hatched juveniles are immediately colonized by the free living *Vibrio* symbionts leading to the maturation of the hosting light organ in parallel with the regression of its ciliated epithelium, preventing latter colonization by other symbionts (Chun et al. 2008). These tissue modifications are triggered by molecules produced by the symbionts which are recognized by the host, hence modifying its transcription patterns. In mammals, during the first phase after birth, the midgut is able to establish a stable host‐bacterial symbiosis that stimulates the intestinal epithelium maturation and also its regeneration (Huang et al. 2013; Hill et al. 2017; Walker 2017; Nigro et al. 2018). Further investigations are required to decipher whether the maturation of the intestinal epithelium of *Rimicaris* shrimps, with the formation of microvilli, is a process entirely controlled by the host cells and is a prerequisite for symbiont colonization, or if this developmental process is triggered by symbionts upon their acquisition.

### 4.2 Acquisition and Transmission of Each Symbiotic Lineage

Our study suggests a fast colonization of symbiotic organs in both *R. exoculata* and *R. chacei* specimens right after their settlement, although with an offset between the primary colonization of digestive symbionts and cephalothoracic symbionts. For the cephalothoracic cavity, the symbiotic relationship is established in all individuals at the earliest juvenile stages. Indeed, at this stage, all the main symbiotic communities colonizing the cephalothoracic cavity of adults are retrieved (*Sulfurovum* spp., *Cocleimonas* spp., *Candidatus* Ghiorsea crypta, *Desulfocapsa* spp.; Zbinden and Cambon Bonavita 2020; Cambon‐Bonavita et al. 2021). The main ASVs affilitated to these bacterial communities are then shared by almost all the specimens whatever the juvenile stage. On the other hand, the digestive symbionts, mostly *Candidatus* Foregutplasma rimicarensis and *Candidatus* Microvillispirillaceae, were only detected in the foregut and the midgut of a few stage A juveniles for both species. A presence of these digestive symbionts in all individuals analyzed was only observed in later stages, from stage B juveniles for *R. exoculata* and from subadults for *R. chacei*. The dominant symbionts are shared by the later stage specimens and are not retrived in the cephalothoracic cavity (except for rare specimens). Therefore, we suggest that symbiont acquisition starts first in the cephalothoracic cavity, right at settlement, and only begins after for the resident digestive symbionts, during the transition between stage A and stage B juveniles for *R. exoculata* and between stage A and subadult juveniles for *R. chacei*.

These observations for the cephalothoracic cavity of juveniles with the presence of all the main symbiotic lineages retrieved in adults (Zbinden and Cambon Bonavita 2020) are in line with previous hypotheses suggesting horizontal transmission of these symbionts. Such transmission mode was strongly suspected for juvenile stages as symbionts are already necessarily renewed at each molt throughout their adult life (Corbari et al. 2008a, 2008b; Cambon‐Bonavita et al. 2021). This is also supported by the fact that the cephalothoracic‐related lineages were retrieved as free‐living in the shrimp environment (Hügler, Gärtner, and Imhoff 2010; Guri et al. 2012; Jan et al. 2014).

Conversely, transmission of the digestive symbionts was less clear. For now, no OTUs related to *Candidatus* Microvillispirillaceae and only one related to *Mycoplasmatales* (order) has been found in hydrothermal fluids around shrimp aggregates (Hügler, Gärtner, and Imhoff 2010; Flores et al. 2011). The data, associated with the presence of one OTU related to *Mycoplasmatales* on a few egg broods of *R. exoculata* (Methou et al. 2019), may have suggested a vertical transmission from mother to embryos at this life stage. However, the results of this study revealed that whatever the site and the shrimp species, the first stage after settlement did not systematically have *Candidatus* Microvillispirillacea nor *Candidatus* Foregutplasma rimicarensis in their digestive system or only a few for some individuals. Consequently, a vertical acquisition at egg stages, maintained all along the lifecycle can be dismissed. A more likely scenario would be that digestive symbionts are acquired horizontally after juvenile settlement. In this case, the apparent absence of the digestive symbionts in the environment may be linked to a spatially restricted niche in specific areas and/or substrates which have not been explored yet. For instance, a presence restricted to nurserie habitats (Methou et al. 2022) or on rocks substrates rather than in the surrounding water could be possible.

As hypothetized by Durand et al. (2015), we also cannot exclude an inter‐generational transmission for digestive symbionts between individuals from adults to juveniles by trophallaxis, or by another mean. In terrestrial isopods, which also host similar lineages of *Hepatoplasmataceae*, horizontal transmission by ingestion of inoculated food sources, either through coprophagy or cannibalism, has been proposed (Bouchon, Zimmer, and Dittmer 2016). The transmission mechanisms of *Candidatus* Foregutplasma rimicarensis and *Candidatus* Microvillispirillaceae might also differ as their hosting organs are submitted to different constraints. *Candidatus* Foregutplasma rimicarensis within the foregut is, for instance, submitted to a renewal of the cuticle at each molt, due to the ectodermic origin of the foregut (Vogt 2021), requiring a renewed acquisition from the environment at each molt. On the other hand, *Candidatus* Microvillispirillacea within the midgut tube (endodermic origin) is not constrained by host exuviation and could be acquired only once during the transition between the settlement stage (juvenile A) and subsequent juvenile stages. Also, the cell division of these lineages appears to be controlled by their shrimp host (Aubé et al. 2022) limiting their proliferation and possibly a release in the environment to colonize other shrimp congeners. Still, this cell division control was observed in adults but could vary during the host life cycle or through molt phases. Indeed, many examples of variations in the host‐symbiont communications and interactions exist, offering possibilities for the symbiont to escape the host's control (Gross et al. 2009; Jacobovitz et al. 2021). So, *Candidatus* Microvillispirillaceae would be ejected from the adult midgut tube with the feces or the cuticle (hindgut) during exuviation and released into the environment. Then, they could be captured by juveniles with the flow of fluids from the posterior (Martin et al. 2020) to proximal regions of the midgut tube.

Still, on an evolutionary time scale, several other evidences are pointing instead towards a vertical transmission of these digestive symbionts, particularly for *Candidatus* Foregutplasma rimicarensis. The presence of related symbiont lineages in alvinocaridid shrimp from other regions but also in crustaceans from other ecosystems (Eberl 2010; Bouchon, Zimmer, and Dittmer 2016; Methou et al. 2023a) was already indicative of an ancient and conserved association, but still insufficient to conclude. However, the highly reduced size of their genomes (0.48–0.83 Mbp for *Hepatoplasmataceae* and 1.25–1.36 Mbp for *Candidatus* Microvillispirillacea) (Aubé et al. 2022), a low GC content and the loss of several essential genes (single copy core genes) clearly suggest a genome reduction which is generally associated with vertical inheritance (a process known as Muller's ratchet, McCutcheon and Moran 2012). For comparison, genome size of symbionts from vesicomyid clams, that follow a strict vertical mode of transmission, are comprised between 1.0 and 1.25 Mbp (Russell et al. 2020). Future investigations of the acquisition of these digestive symbionts in other alvinocaridids, or in other crustaceans for the case of *Candidatus* Foregutplasma rimicarensis, should help to clarify these apparent discrepancies between our ecological observations and the evolutionary patterns.

### 4.3 Dynamic of Symbiotic Communities Along the Post‐Settlement Metamorphosis

*Candidatus* Foregutplasma rimicarensis and *Candidatus* Bg2_rimicarensis, i.e., the main colonists of the foregut, were also detected in the midgut tube of older juveniles (stage B and subadults) in both *Rimicaris* species. However, in adults, these symbiotic lineages are known to be more abundant in the foregut (Aubé et al. 2022; Guéganton et al. 2022). This could reflect an initial colonization of the entire digestive system and not specifically of the foregut at juvenile and subadult stages. Such colonization process has similarities with what is observed in the tubeworm *Riftia pachyptila* which endosymbiotic bacteria are also acquired after settlement, at the post‐larval stage. At the adult stage their symbionts are hosted in a dedicated organ—the trophosome—but during the acquisition phase, they colonize all host tissues after entering through the worm epidermis, before a migration at later life stages towards the tissue layer that give birth to the adult trophosome (Nussbaumer, Fisher, and Bright 2006). Even with a constant symbiont acquisition throughout their adult life (Wentrup et al. 2014), a similar phenomenon has also been observed in bathymodiolin mussels with a first colonization by symbiotic lineages between the pediveliger and metamorphosis stages in all of their organs before being restricted to gill tissues at older stages (Wentrup et al. 2013; Franke et al. 2021). Similarly, in *Rimicaris* shrimp from the MAR, the compartimentalization of *Candidatus* Foregutplasma rimicarensis and *Candidatus* Bg2_rimicarensis symbionts only within their foregut could be acquired progressively and finalized after the metamorphosis with an aspecific colonization of both organs at juvenile and subadult stages. On the other hand, the presence of *Candidatus* Foregutplasma rimicarensis and *Candidatus* Bg2_rimicarensis as *Sulfurovum* spp. in the midgut tube could be explained by molt ingestion. As a consequences, theses lineages would be considered as no symbiotic and would go through the alimentary bolus (no visible on tissues with FISH microscopy). This hypothesis could also explain the fact that as *Sulfurovum* spp. are also retrived in the foregut.

Interestingly, in *Rimicaris variabilis* and *Nautilocaris saintlaurentae*, two alvinocaridid shrimp that host similar lineages of digestive symbionts, both *Candidatus* Microvillispirillaceae and *Candidatus* Foregutplasma rimicarensis symbionts were found within the foregut and the midgut even at adult stages, suggesting that compartmentalization of these symbionts might exist in some alvinocaridids but not all (Methou et al. 2023a). Potentially, this could be due to differences in their diets with an organ partitioning of digestive symbionts in species relying, at least in part, on their cephalothoracic symbiosis—i.e., *R. exoculata* and *R. chacei*—and an aspecific colonization in species relying on a bacterivory/scavenging diet—i.e., *R. variabilis* and *N. saintlaurentae*—even if a wider comparison including more alvinocaridid species would be required to confirm this pattern. At last, we cannot exclude either the hypothesis of contamination during our dissections (as for *Candidatus* Microvillispirillaceae retrieved in two subsault foregut specimens), or leakage of stomach bacteria towards the midgut tube due to animal stress during their sampling.

### 4.4 Variability of Symbiotic Communities Among Host Species and Vent Sites

At equivalent stage, the cephalothoracic cavity was more colonized in *R. exoculata* than in *R. chacei*. Indeed, even at the subadult stage, branchiostegites of *R. chacei* were barely colonized, contrary to the mouthparts which were well colonized by a complex and mature symbiotic community including among others, *Cocleimonas* spp. (*Gammaproteobacteria* class) and *Sulfurovum* spp. (*Campylobacteria* class) filamentous bacteria (Apremont et al. 2018). This contrasts with the dense bacterial colonization observed on both mouthparts and branchiostegites of *R. exoculata* already observed in stage B juveniles. Even stage A juveniles of *R. exoculata* exhibit denser bacterial colonization in the cephalothoracic cavity than stage A of *R. chacei*. This is in line with the gradual transitions towards distinct diets of the two species during their post‐settlement metamorphosis, respectively to a chemosymbiotic diet for *R. exoculata* and to a mixotrophic behavior for *R. chacei* (Methou et al. 2020). These differences in the colonization patterns of the two species as well as in their trophic transitions are also reflected at the anatomical level during this post‐settlement metamorphosis phase. As evidenced by Methou et al. (2023b) and our observations, enlargement of the mouthparts and the branchiostegites is more marked for *R. exoculata* whereas foregut size exhibited a drastic increase in *R. chacei*. Just as the appearance of microvilli in the digestive tract of juveniles was concomitant with the symbiotic colonization of this organ, it is interesting to see that the absence of branchiostegite enlargement in *R. chacei* coincides with the absence of colonization of this anatomical structure in subadults. Conversely, complete colonization of these branchiostegites at an equivalent stage in the genetically similar shrimp *R. hybisae* gives rise to significant hypertrophy of their cephalothoracic cavity at adult stages (Methou et al. 2023b). Although a causal link cannot be clearly established yet, all these observations question the respective role of the symbionts and their host in the developmental processes during the metamorphosis phase of these shrimp.

The symbiotic communities hosted in both *Rimicaris* spp. seem to also be influenced by the vent field of origin and could be related to known differences in the fluid composition of TAG and Snake Pit (Fouquet et al. 2010). In the cephalothoracic cavity, the same dominant symbiotic lineage is present at each stage for both species (*Sulfuvorum* spp.) but variations are visible for other lineages, as seen in adult stages or on the surface of eggs (Methou et al. 2019; Cambon‐Bonavita et al. 2021). For instance, the iron‐oxidizing *Candidatus* Ghiorsea crypta (*Zetaproteobacteria* class) are much more abundant at TAG and almost absent at Snake Pit which can be linked to the higher iron concentration in TAG vent fluids (Fouquet et al. 2010).

## 5 Conclusion

Upon settlement on sites, juveniles of each *Rimicaris* species occupy a different habitat, which is adjacent to adults for *R. exoculata* but on diffusing areas without adults for *R. chacei*. Such spatial segregation could have an impact for the acquisition of symbionts in each species. Overall, our data do not support previous hypotheses predicting vertical transmission for some symbiont lineages, but rather highlight an horizontal symbiont acquisition from local bacterial pools following the dispersal phase both for the cephalothoracic and digestive symbioses. However, different pathways of transmission could occur for each symbioses. Cephalothoracic symbionts are likely acquired from an environmental free‐living pool, while digestive symbionts, showing streamlined genomes, probably also involve specific mechanisms of symbiont release from older stages with established symbiosis allowing colonization of early juveniles. The two symbioses also differ by their colonization dynamic with a cephalothoracic cavity rapidly colonized with diverse bacterial lineages present from the earliest juvenile stages, while digestive systems seem to develop afterwards with only well established symbiont communities at later juvenile stages. The colonization of symbionts in the cephalothoracic cavity is also different for the two host species. Whatever the stage, *R. exoculata* juveniles harbor a denser bacterial colonization than *R. chacei* juveniles. This difference may be in line with the absence of branchiostegite enlargement in *R. chacei* and with the habitat. This difference in colonization could potentially explain, at term, the collapse of *R. chacei* population during recruitment.

Still, larval stages remain a major gap to fully understand acquisition of symbiotic lineages all along the host life cycle and confirm if these life stages are aposymbiotic. However technical difficulties to sample or to rear larvae at laboratory preclude detailed study of the larval biology of these shrimp at the moment.

## Author Contributions

**Marion Guéganton:** data curation (lead), formal analysis (lead), investigation (lead), methodology (lead), software (equal), visualization (lead), writing – original draft (lead), writing – review and editing (lead). **Pierre Methou:** resources (equal), supervision (equal), validation (lead), writing – review and editing (lead). **Johanne Aubé:** formal analysis (lead), software (lead), validation (equal), writing – review and editing (equal). **Cyril Noël:** software (lead), writing – review and editing (supporting). **Ouafae Rouxel:** investigation (equal), methodology (equal), writing – review and editing (supporting). **Valérie Cueff‐Gauchard:** resources (lead), software (equal), writing – review and editing (supporting). **Nicolas Gayet:** investigation (equal), writing – review and editing (supporting). **Lucile Durand:** investigation (supporting), methodology (equal), writing – review and editing (supporting). **Florence Pradillon:** conceptualization (lead), resources (equal), supervision (lead), validation (lead), writing – review and editing (lead). **Marie‐Anne Cambon‐Bonavita:** conceptualization (lead), funding acquisition (lead), project administration (lead), resources (lead), supervision (lead), validation (lead), writing – review and editing (lead).

## Conflicts of Interest

The authors declare no conflicts of interest.

## Supporting information


Figures S1–S5

**Table S1.** α diversity and β diversity: Direct vs Nested PCR.
**Table S2.** ANCOM‐BC2 on ASVs according to the genus (PCR).
**Table S3.** ANCOM‐BC2 on ASVs according to the family (PCR).
**Table S4.** Decontaminated ASVs table (all organs).
**Table S5.** α diversity and β diversity: Total juveniles.
**Table S6.** α diversity and β diversity: Foregut.
**Table S7.** ANCOM‐BC2 on ASVs according to the stage (foregut).
**Table S8.** α diversity and β diversity: Midgut tube.
**Table S9.** ANCOM‐BC2 on ASVs according to the stage (midgut tube).
**Table S10.** α diversity and β diversity: Cephalothoracic cavity.
**Table S11.** ANCOM‐BC2 on ASVs according to the species (cephalothoracic cavity).
**Table S12.** ANCOM‐BC2 on ASVs according to the site (cephalothoracic cavity).
**Table S13.** Metabarcoding Metadata.

Appendices S1–S8

## Data Availability

The raw reads of the metabarcoding dataset are available in the European Nulceotide Archive under Bioproject Accession Number PRJEB71821. The configuration files for the processes options used in SAMBA are avalaible in the Appendices S7 and S8. The sequences of COI are available on NCBI under accession numbers PP916018‐PP916042 (Table S13).
