# Supplementary material for: Symbiont Acquisition Strategies in Post‐Settlement Stages of Two Co‐Occurring Deep‐Sea Rimicaris Shrimp
Source: Ecol Evol. 2024 Nov 19;14(11):e70369. doi: 10.1002/ece3.70369 (PMC11576329; doi:10.1002/ece3.70369)
Supplement: Supplementary file 1 — Figures S1–S5 [file ECE3-14-e70369-s002.pdf]

Supplementary Figures: Symbiont acquisition strategies in post-settlement stages of two co-occurring deep-sea *Rimicaris* shrimp.

Supplementary Figure 1:

PCR comparison results. (A) Bacterial taxonomic composition at the genus-level grouped by PCR approaches of juvenile A *R. exoculata* symbiotic tissues through direct (9 right hand side bars) and nested PCR (9 left hand side bars in the same order than the 9 on the right) same sample in the same order. **(B)** NMDS plot of Jaccard distance between the bacterial diversity amplified samples, colored according to the PCR used. Ellipses represent 95% confidence interval for each group. G : Gills; CEP : Cephalothoracic cavity; MG : Midgut tube; FG : Foregut.

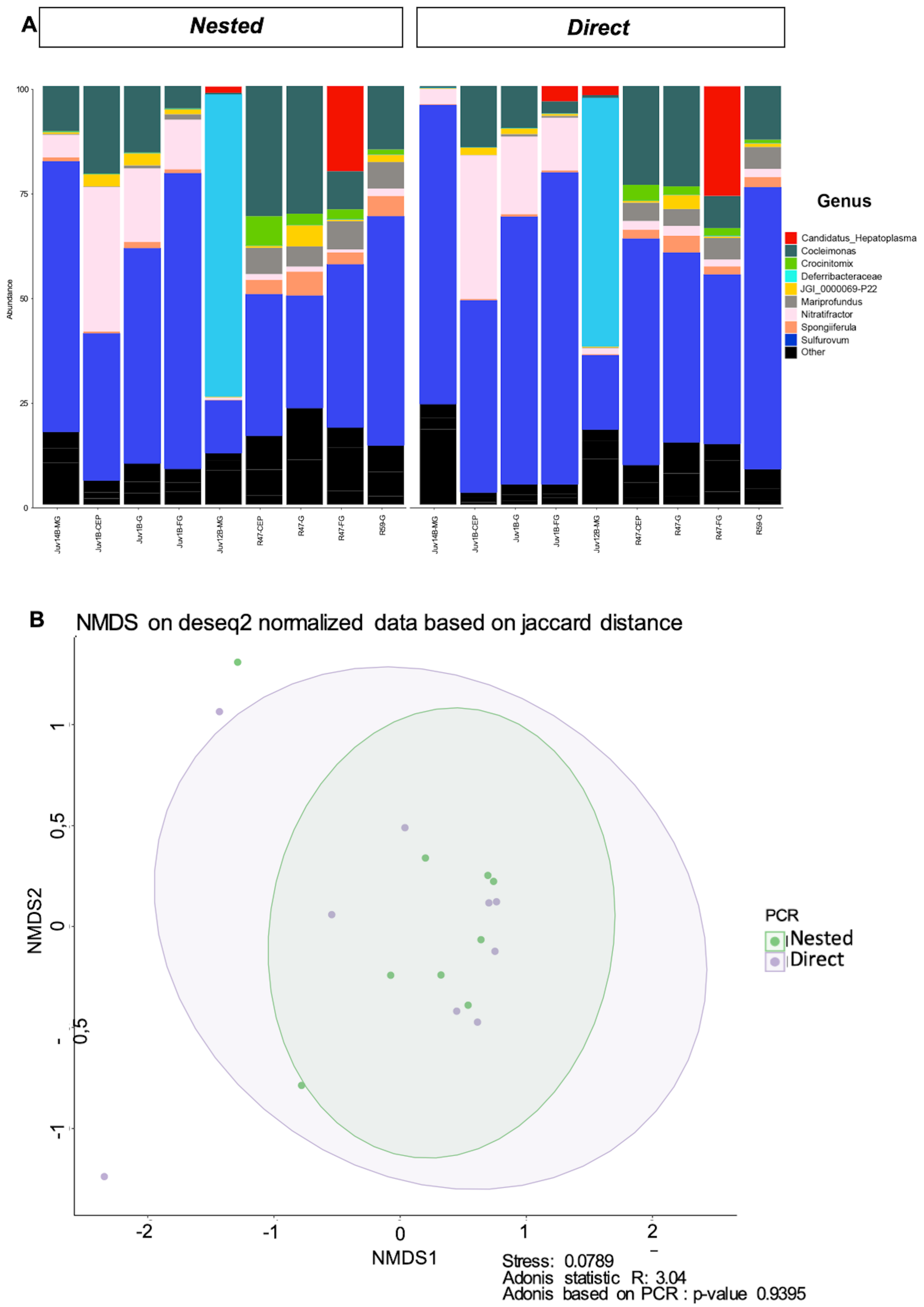
A
Nested
Direct
Abundance
100
75
50
25
0
Juv14B-MG
Juv1B-CEP
Juv1B-G
Juv1B-FG
Juv12B-MG
R47-CEP
R47-G
R47-FG
R59-G
Genus
Candidatus_Hepatoplasma
Cocleimonas
Crocinitomix
Deferribacteraceae
JGI_0000069-P22
Mariprofundus
Nitratifractor
Spongiiferula
Sulfurovum
Other
B
NMDS on deseq2 normalized data based on jaccard distance
NMDS2
NMDS1
PCR
Nested
Direct
Stress: 0.0789
Adonis statistic R: 3.04
Adonis based on PCR : p-value 0.9395

12

Supplementary Figure 2:

More details on the structures of the cardiac and pyloric chambers of *R. exoculata* subadult specimen with Scanning Electron Microscopy and beta diversity. (A) General structure of the cardiac and pyloric chambers in ventral view. Black squares represent a focus on the cardiac floor crest and on the diverse elements of the pyloric chamber. **(B)** General structure of the cardiac and pyloric chambers in dorsal view. Black squares represent a focus on the cardiac floor crest and on the diverse elements of the pyloric chamber**. (C)** Dorsal view of the cardiac floor crest with its setae (serrulate setae, setae of the unpaired anterior ossicles). **(D)** Pyloric filter with the setae of the ossicle' layers. **(E)** Setae of the lateral walls of the pyloric chamber. **(F)** Setae of the dorsal side of the pyloric chamber.

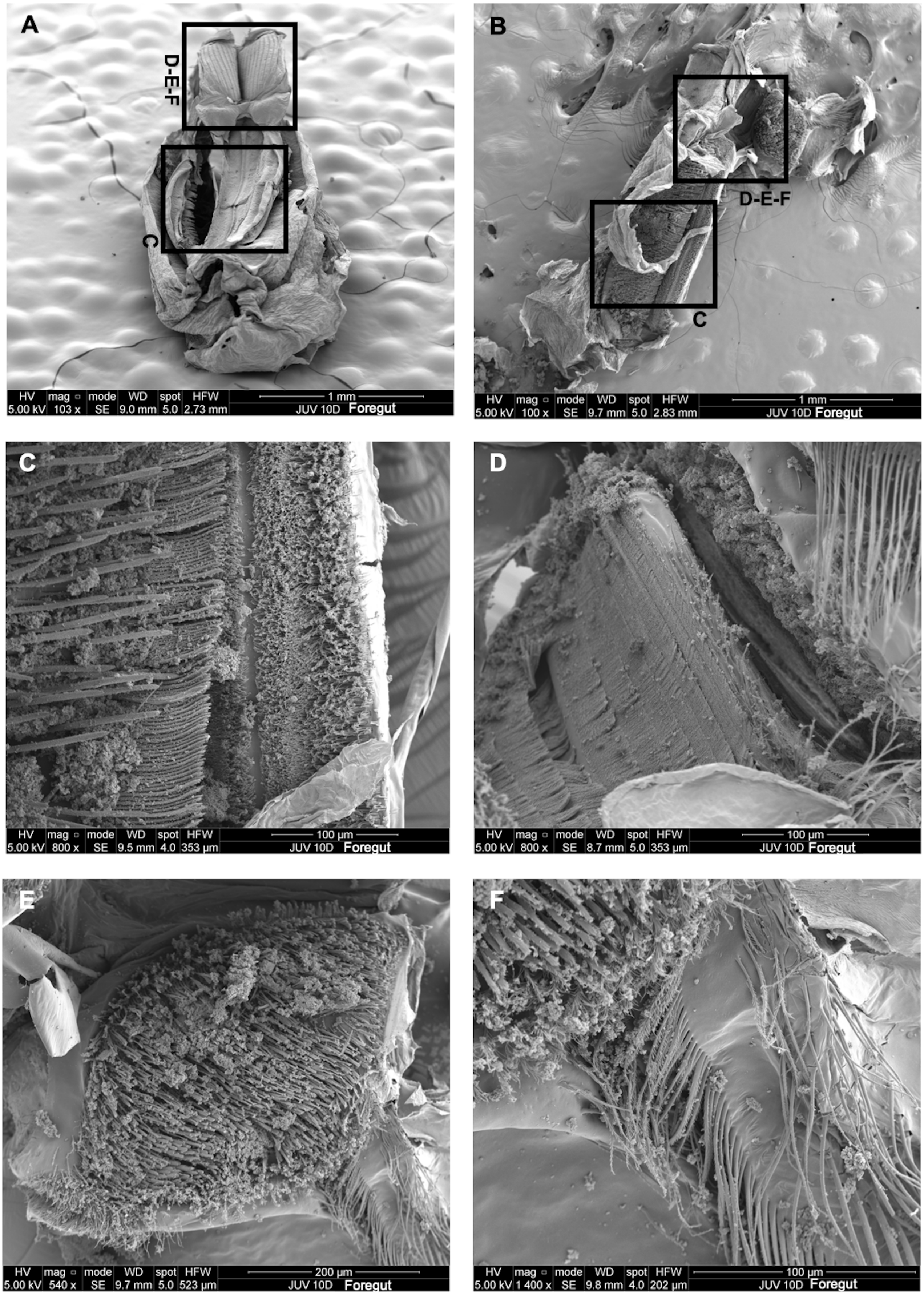
A
D-E-F
C
HV mag mode WD spot HFW
5.00 kV 103 x SE 9.0 mm 5.0 2.73 mm
1 mm
JUV 10D Foregut
B
D-E-F
C
HV mag mode WD spot HFW
5.00 kV 100 x SE 9.7 mm 5.0 2.83 mm
1 mm
JUV 10D Foregut
C
HV mag mode WD spot HFW
5.00 kV 800 x SE 9.5 mm 4.0 353 µm
100 µm
JUV 10D Foregut
D
HV mag mode WD spot HFW
5.00 kV 800 x SE 8.7 mm 5.0 353 µm
100 µm
JUV 10D Foregut
E
HV mag mode WD spot HFW
5.00 kV 540 x SE 9.7 mm 5.0 523 µm
200 µm
JUV 10D Foregut
F
HV mag mode WD spot HFW
5.00 kV 1 400 x SE 9.8 mm 4.0 202 µm
100 µm
JUV 10D Foregut

Supplementary Figure 3:

NMDS plots of Jaccard distance according to the species and life stage. (A) NMDS plot of Jaccard distance between foregut samples colored according to the species. Stress : 0.1597; Adonis statistic R : 7.99; Adonis based on species : p-value 1e-04.**(B)** NMDS plot of Jaccard distance between foregut samples colored according to the life stages. Stress : 0.1597; Adonis statistic R : 5.91; Adonis based on stage : p-value 0.0083. **(C)** NMDS plot of Jaccard distance between midgut tube samples colored according to the species. Stress : 0.2031; Adonis statistic R : 5.77; Adonis based on species : p-value 1e-04. **(D)** NMDS plot of Jaccard distance between midgut tube samples colored according to the life stages. Stress : 0.2031; Adonis statistic R : 6.06; Adonis based on stage : p-value 5e-04. **(E)** NMDS plot of Jaccard distance between cephalothorax samples colored according to the species. Stress : 0.1293; Adonis statistic R : 11.34; Adonis based on species : p-value 1e-04. **(F)** NMDS plot of Jaccard distance between cephalothorax samples colored according to the life stages. Stress : 0.1293; Adonis statistic R : 5.73; Adonis based on stage : p-value 0.0457.Ellipses represent 95% confidence interval for each group.

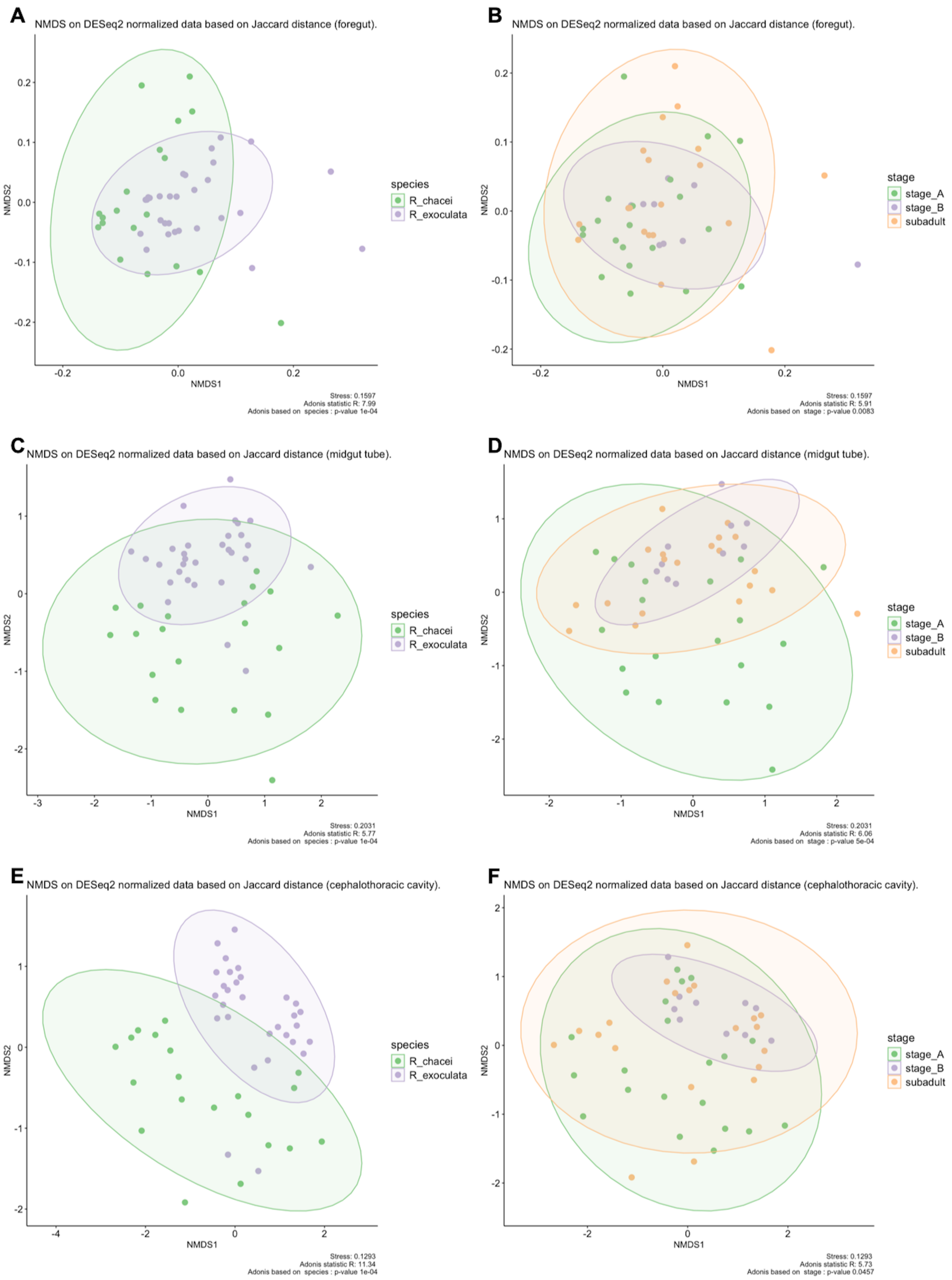
A
NMDS on DESeq2 normalized data based on Jaccard distance (foregut).
NMDS2
NMDS1
species
R_chacei
R_exoculata
Stress: 0.1597
Adonis statistic R: 7.99
Adonis based on species : p-value 1e-04
B
NMDS on DESeq2 normalized data based on Jaccard distance (foregut).
NMDS2
NMDS1
stage
stage_A
stage_B
subadult
Stress: 0.1597
Adonis statistic R: 5.91
Adonis based on stage : p-value 0.0083
C
NMDS on DESeq2 normalized data based on Jaccard distance (midgut tube).
NMDS2
NMDS1
species
R_chacei
R_exoculata
Stress: 0.2031
Adonis statistic R: 5.77
Adonis based on species : p-value 1e-04
D
NMDS on DESeq2 normalized data based on Jaccard distance (midgut tube).
NMDS2
NMDS1
stage
stage_A
stage_B
subadult
Stress: 0.2031
Adonis statistic R: 6.06
Adonis based on stage : p-value 5e-04
E
NMDS on DESeq2 normalized data based on Jaccard distance (cephalothoracic cavity).
NMDS2
NMDS1
species
R_chacei
R_exoculata
Stress: 0.1293
Adonis statistic R: 11.34
Adonis based on species : p-value 1e-04
F
NMDS on DESeq2 normalized data based on Jaccard distance (cephalothoracic cavity).
NMDS2
NMDS1
stage
stage_A
stage_B
subadult
Stress: 0.1293
Adonis statistic R: 5.73
Adonis based on stage : p-value 0.0457

53

Supplementary Figure 4:

More details on the midgut tube with Scanning Electron Microscopy (A, B and C), Fluorescent *in situ* Hybridization (D and F) and beta diversity (C). (A) Microvilli (white arrow) observed in the midgut tube of a *R. exoculata* stage B. **(B)** Microvilli (white arrow) observed in the midgut tube of a *R. chacei* subadult. **(C)** Microvilli (white arrow) observed in the midgut tube of a *R. exoculata* subadult. **(D)** Autofluorescence of minerals contained in the alimentary bolus was observed in the midgut tube of a *R. exoculata* stage A from TAG (yellow and red). **(E)** *Candidatus* Microvillispirillaceae of the midgut tube with the alimentary bolus of a *R. chacei* subadult from Snake Pit co-hybridized with the specific probes Def1229-Cy3 and Eub338-Cy5 (yellow, light-orange). Autofluorescence of minerals contained in the alimentary bolus was observed in yellow and red. **(F)** *Candidatus* Microvillispirillaceae of the midgut tube of a *R. exoculata* stage B from Snake Pit hybridized with the specific probe Def1229-Cy3 (orange). **(D-F)** Tissue cell nuclei are labelled with DAPI (blue). Scale bars = 20 µm.

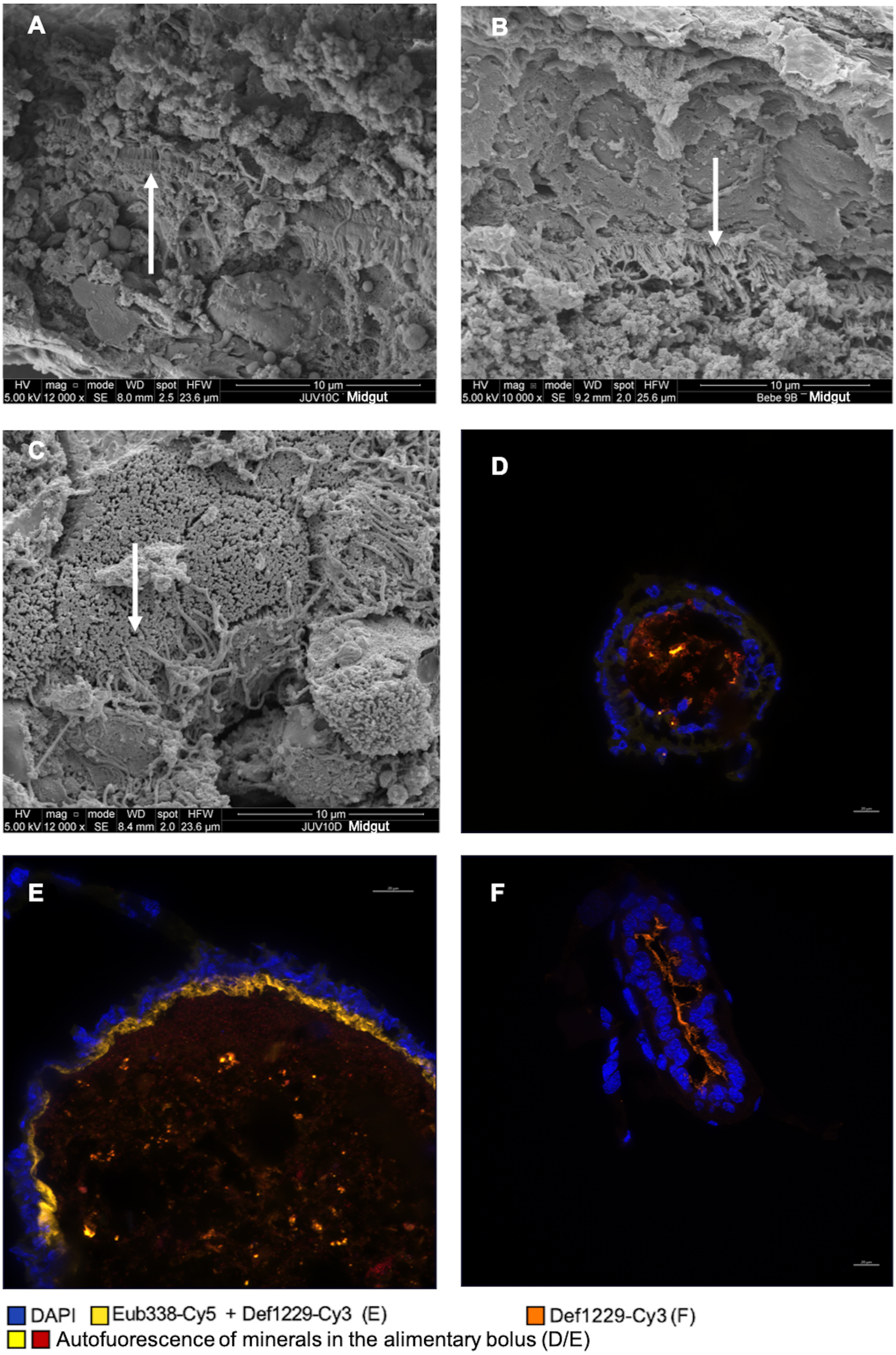
A
HV mag mode WD spot HFW
5.00 kV 12 000 x SE 8.0 mm 2.5 23.6 µm
10 µm
JUV10C Midgut
B
HV mag mode WD spot HFW
5.00 kV 10 000 x SE 9.2 mm 2.0 25.6 µm
10 µm
Bebe 9B Midgut
C
HV mag mode WD spot HFW
5.00 kV 12 000 x SE 8.4 mm 2.0 23.6 µm
10 µm
JUV10D Midgut
D
E
F
DAPI
Eub338-Cy5 + Def1229-Cy3 (E)
Def1229-Cy3 (F)
Autofuorescence of minerals in the alimentary bolus (D/E)

70

Supplementary Figure 5:

More details on the cephalothoracic symbiosis. Using FISH, *Gammaproteobacteria* were hybridized with the specific probe GAM42a, *Campylobacterales* with Epsy549, *Zetaproteobacteria* with Zeta709, *Bacteroidia* with CF319a and *Desulfobulbales* with DSB706. **(A)** Bacteria on setae of a *R. chacei* subadult from Snake Pit hybridized with the specific Epsy549-ATTO488 (green), GAM42a-Cy5 (red) and CF319a-Cy3 (yellow). **(B)** Bacteria on scaphognathites of a *R. chacei* subadult from Snake Pit hybridized with the specific Epsy549-ATTO488 (green), GAM42a-Cy5 (red) and CF319a-Cy3 (yellow). **(C)** Bacteria on branchiostegites of a *R. exoculata* subadult from Snake Pit hybridized with the specific probes Epsy549-ATTO488 (green), GAM42a-Cy5 (red) and DSB706-Cy3 (yellow). **(D)** Bacteria in the cephalothoracic cavity of a *R. chacei* subadult from Snake Pit hybridized with the specific probes Epsy549-ATTO488 (blue-green), GAM42a-Cy5 (red) and Zeta709-Cy3 (yellow).). **(B-D)** Tissue cell nuclei were labelled with DAPI (blue). Scale bars = 20 µm.

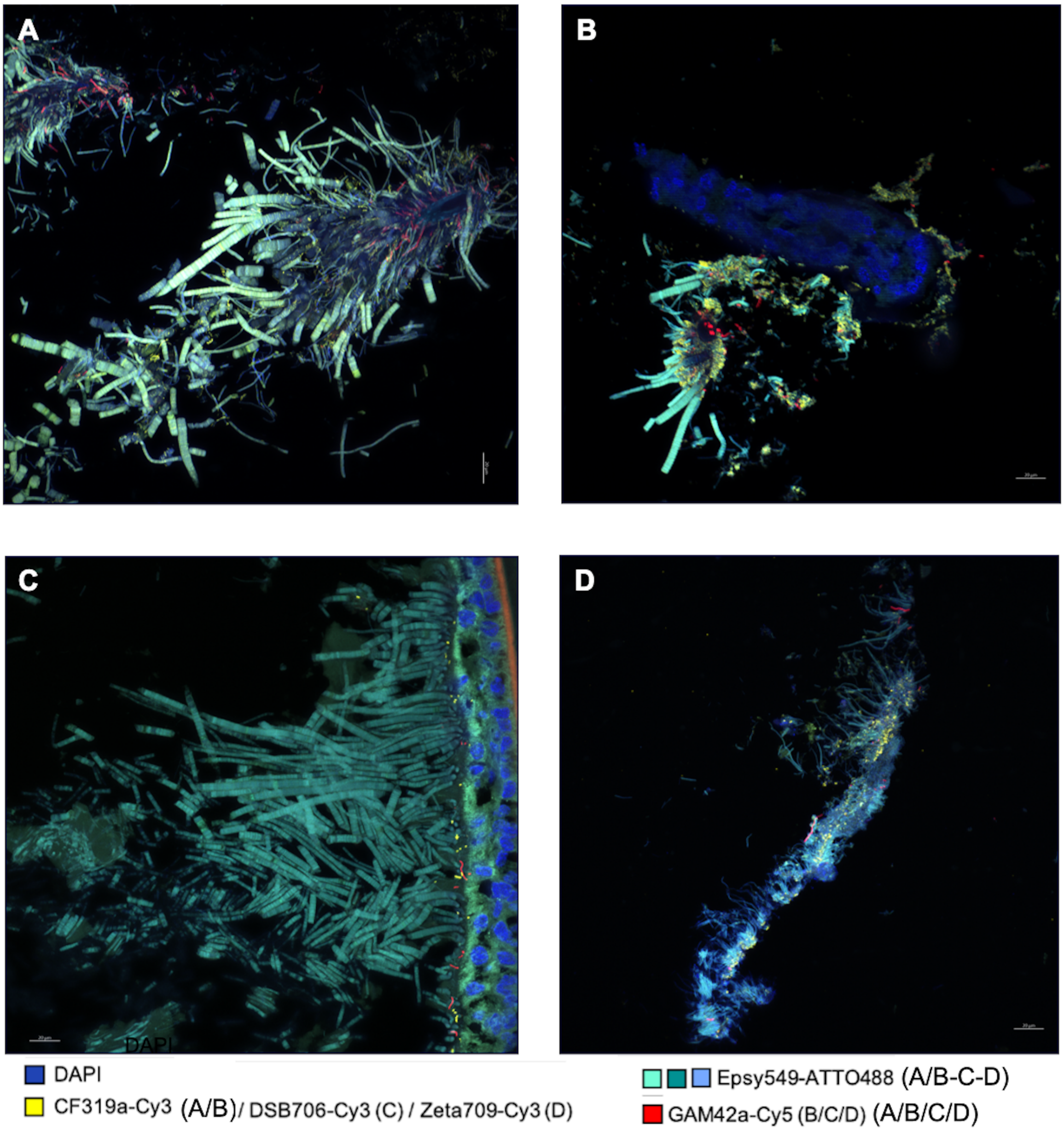
A
B
C
D
DAPI
CF319a-Cy3 (A/B)/ DSB706-Cy3 (C) / Zeta709-Cy3 (D)
Epsy549-ATTO488 (A/B-C-D)
GAM42a-Cy5 (B/C/D) (A/B/C/D)
