# Supplementary material for: Symbiont Acquisition Strategies in Post‐Settlement Stages of Two Co‐Occurring Deep‐Sea Rimicaris Shrimp
Source: Ecol Evol. 2024 Nov 19;14(11):e70369. doi: 10.1002/ece3.70369 (PMC11576329; doi:10.1002/ece3.70369)
Supplement: Supplementary file 3 — Appendices S1–S8 [file ECE3-14-e70369-s003.docx]

# Appendix: Symbiont acquisition strategies in post-settlement stages of two co-occurring deep-sea *Rimicaris* shrimp.

### Appendix 1:

**Global view of the specimens used for FISH, SEM and metabarcoding studies**

| Species (life stage)/site | **FISH** | **Metabarcoding** | **SEM** |
| --- | --- | --- | --- |
| *R. exoculata* (stage A)/ TAG | 3 specimens | 5 specimens | 1 specimen |
| *R. exoculata* (stage B)/ TAG | 3 specimens | 5 specimens | 1 specimen |
| *R. exoculata* (subadult)/ TAG | 3 specimens | 5 specimens | 1 specimen |
| *R. exoculata* (stage A)/ Snake Pit | 4 specimens | 5 specimens |  |
| *R. exoculata* (stage B)/ Snake Pit | 3 specimens | 5 specimens |  |
| *R. exoculata* (subadult)/ Snake Pit | 2 specimens | 5 specimens |  |
| *R. chacei* (stage A)/ TAG | 3 specimens | 5 specimens | 1 specimen |
| *R. chacei* (subadult)/ TAG | 3 specimens | 5 specimens | 1 specimen |
| *R. chacei* (stage A)/ Snake Pit | 2 specimens | 5 specimens |  |
| *R. chacei* (subadult)/ Snake Pit | 3 specimens | 5 specimens |  |

### Appendix 2:

**Juveniles used for symbiont metabarcoding, showing their stage, origin, and for each tissue, the symbiont rRNA gene amplification procedure**. CEP: cephalothoracic cavity; G: gills; FG: Foregut; MG: Midgut tube; N: Nested PCR; D: Direct PCR.

| **ID** | **COI** | **Stage** | **Site** | **Tissue** | **PCR** |
| --- | --- | --- | --- | --- | --- |
| **Cr108** | *R. chacei* | A | TAG | CEP/G/FG/MG | N/N/N/N |
| **bebe12A** | *R. chacei* | A | TAG | CEP/G/FG/MG | D/N/N/D |
| **Cr107** | *R. chacei* | A | TAG | CEP/G/FG/MG | N/N/N/N |
| **R08** | *R. chacei* | A | TAG | CEP/G/FG/MG | D/N/N/D |
| **R32** | *R. chacei* | A | TAG | CEP/G/FG/MG | D/N/N/D |
| **bebe13A** | *R. chacei* | A | Snake Pit | CEP/G/FG/MG | N/N/N/N |
| **bebe15A** | *R. chacei* | A | Snake Pit | CEP/G/FG/MG | N/D/N/N |
| **bebe24A** | *R. chacei* | A | Snake Pit | CEP/G/FG/MG | N/N/N/N |
| **CR011** | *R. chacei* | A | Snake Pit | CEP/G/FG/MG | N/N/D/N |
| **Cr051** | *R. chacei* | A | Snake Pit | CEP/G/FG/MG | D/D/N/N |
| **bebe1B** | *R. chacei* | Subadult | TAG | CEP/G/FG/MG | N/N/N/N |
| **bebe2B** | *R. chacei* | Subadult | TAG | CEP/G/FG/MG | D/D/D/N |
| **bebe3B** | *R. chacei* | Subadult | TAG | CEP/G/FG/MG | D/N/N/N |
| **R26** | *R. chacei* | Subadult | TAG | CEP/G/FG/MG | D/D/N/N |
| **R39** | *R. chacei* | Subadult | TAG | CEP/G/FG/MG | D/N/N/N |
| **bebe16B** | *R. chacei* | Subadult | Snake Pit | CEP/G/FG/MG | N/N/N/N |
| **bebe17B** | *R. chacei* | Subadult | Snake Pit | CEP/FG/MG | N/N/N |
| **bebe24B** | *R. chacei* | Subadult | Snake Pit | CEP/G/MG | N/D/N |
| **bebe25B** | *R. chacei* | Subadult | Snake Pit | CEP/G/FG/MG | D/D/N/N |
| **bebe24C** | *R. chacei* | Subadult | Snake Pit | CEP/G/FG/MG | D/D/N/N |
| **Juv12B** | *R. exoculata* | A | TAG | CEP/G/FG/MG | D/N/N/D |
| **Juv1B** | *R. exoculata* | A | TAG | CEP/G/FG/MG | D/D/D/N |
| **R138** | *R. exoculata* | A | TAG | CEP/G/FG/MG | D/D/D/N |
| **R47** | *R. exoculata* | A | TAG | CEP/G/FG/MG | D/D/D/N |
| **R59** | *R. exoculata* | A | TAG | CEP/G/FG/MG | D/D/D/N |
| **Cr040** | *R. exoculata* | A | Snake Pit | CEP/G/FG/MG | D/N/D/N |
| **Juv14B** | *R. exoculata* | A | Snake Pit | CEP/G/FG/MG | D/D/D/D |
| **Juv15B** | *R. exoculata* | A | Snake Pit | CEP/G/FG/MG | D/D/D/N |
| **Juv16B** | *R. exoculata* | A | Snake Pit | CEP/G/FG/MG | D/D/N/N |
| **Juv17B** | *R. exoculata* | A | Snake Pit | CEP/FG/MG | D/D/N |
| **Juv12C** | *R. exoculata* | B | TAG | CEP/G/FG/MG | D/D/D/D |
| **Juv2C** | *R. exoculata* | B | TAG | CEP/G/FG/MG | D/D/D/D |
| **Juv4C** | *R. exoculata* | B | TAG | CEP/G/FG/MG | D/D/D/D |
| **R185** | *R. exoculata* | B | TAG | CEP/G/FG/MG | D/D/N/D |
| **R61** | *R. exoculata* | B | TAG | CEP/G/FG/MG | D/D/D/D |
| **Cr047** | *R. exoculata* | B | Snake Pit | CEP/G/FG/MG | D/D/N/D |
| **Cr048** | *R. Exoculata* | B | Snake Pit | CEP/MG | D/N |
| **Juv14C** | *R. exoculata* | B | Snake Pit | CEP/G/FG/MG | D/D/D/N |
| **Juv15C** | *R. exoculata* | B | Snake Pit | CEP/G/FG/MG | D/D/D/N |
| **Juv16C** | *R. exoculata* | B | Snake Pit | CEP/FG/MG | D/N/N |
| **Cr178** | *R. exoculata* | Subadult | TAG | CEP/FG/MG | D/D/D |
| **Cr179** | *R. exoculata* | Subadult | TAG | CEP/G/FG/MG | D/N/D/D |
| **Cr180** | *R. exoculata* | Subadult | TAG | CEP/G/FG/MG | D/D/D/D |
| **Cr186** | *R. exoculata* | Subadult | TAG | CEP/G/FG/MG | D/D/D/D |
| **Juv3D** | *R. exoculata* | Subadult | TAG | CEP/G/FG/MG | D/D/D/D |
| **Juv17D** | *R. exoculata* | Subadult | Snake Pit | CEP/G/FG/MG | D/D/D/N |
| **R254** | *R. exoculata* | Subadult | Snake Pit | CEP/G/FG/MG | D/D/D/D |
| **R261** | *R. exoculata* | Subadult | Snake Pit | CEP/G/FG/MG | D/D/D/D |
| **R271** | *R. exoculata* | Subadult | Snake Pit | CEP/G/FG/MG | D/N/N/N |
| **R272** | *R. exoculata* | Subadult | Snake Pit | CEP/G/FG/MG | D/D/N/N |
| **Nested PCR blank** |  |  |  | T2n1/2/3/4/5 | D and N |
| **PCR blank** |  |  |  | T2/ Tn4 | D/D |

### Appendix 3:

#### COI Sequencing

Amplification conditions were as follows: a 5 minutes denaturation step at 95°C, then 35 cycles including 1 minute at 95°C (denaturation), 1 minute at 50°C (annealing) and 2 minutes at 72°C (extension), and a final elongation step of 7 minutes at 72°C. The PCR reaction mix (50 µL) was composed of 1X Taq Buffer (Green GoTaq® Flexi buffer, Promega), 2mM MgCl_2_ (Promega), 0.8 mM dNTP mix (MP Biomedicals™, LLC), 0,3 µM of each primer (Eurogentec, Liege, Belgium), 1,25 U of the Taq Polymerase (GoTaq^®^ G2 Hot Start Polymerase, Promega) and 2 µL of pervious extracted DNA template.

#### Direct PCR

Amplification conditions were as follows: 5 minutes at 94°C, then 30 cycles including 1 minute at 94°C, 1 minute at 52°C and 1 minute 30 seconds at 72°C, and a final step of 6 minutes at 72°C. PCR reaction mix was composed of 1X Taq Buffer (10X Incubation Mix with MgCl_2_, MP Biomedicals™, LLC), 0.2 mM dNTP mix (MP Biomedicals™, LLC), 0,2 µM of each primer (Eurogentec, Liege, Belgium), 2,5 U of the Taq DNA Polymerase (Taq Core, MP Biomedicals™, LLC) and respectively 1 µL, 2 µL and 2 µL diluted 1/10 of DNA template.

#### Nested PCR

The first amplification conditions were as follows: 5 minutes at 95°C, then 30 cycles including 1 minute at 94°C, 1 minute 30 seconds at 49°C and 2 minutes at 72°C, and a final step of 6 minutes at 72°C. PCR reaction was composed of 1X Taq Buffer (10X Incubation Mix with MgCl_2_, MP Biomedicals™, LLC), 0.2 mM dNTP mix (MP Biomedicals™, LLC), 0,3 µM of each primer (Eurogentec, Liege, Belgium), 1,25 U of the Taq DNA Polymerase (Taq Core, MP Biomedicals™, LLC) and respectively 1 µL, 2 µL and 2 µL diluted 1/10 of DNA template.

The second amplification conditions were as follows: 5 minutes at 94°C, then 20 cycles (to limit amplification of contaminants) including 1 minute at 94°C, 1 minute at 53°C and 1 minute 30 seconds at 72°C, and a final step of 6 minutes at 72°C. The PCR reaction was composed of 1X Taq Buffer (10X Incubation Mix with MgCl_2_, MP Biomedicals™, LLC), 0.2 mM dNTP mix (MP Biomedicals™, LLC), 0,2 µM of each primer (Eurogentec, Liege, Belgium), 2,5 U of the Taq DNA Polymerase (Taq Core, MP Biomedicals™, LLC) and 1 µL of amplicons from the first PCR.

### Appendix 4:

**Slides used for FISH observations.**

| ***Slides for FISH*** |  |  |  |  |  |  |  |
| --- | --- | --- | --- | --- | --- | --- | --- |
|  |  |  |  |  |  |  |  |
|  | ***TAG / Stage A*** | ***TAG / Stage B*** | ***TAG / Subadult*** | ***Snake Pit / Stage A*** | ***Snake Pit / Stage B*** | ***Snake Pit / Subadult*** | ***Number of section per slides*** |
| ***R. exoculata (entire)*** | 1 |  | 13 | 45 |  |  | 3 to 4 |
| ***R. exoculata (Branchiostegite)*** | 4 | 8 | 8 | 8 | 8 | 14 | 4 to 6 |
| ***R. exoculata (Scaphognathites)*** | 4 | 8 | 8 | 8 | 8 | 14 | 4 to 6 |
| ***R. exoculata (Exopodites)*** | 4 | 8 | 8 | 8 | 8 | 14 | 4 to 6 |
| ***R. chacei (entire)*** | 14 |  | 9 |  |  |  | 3 to 4 |
| ***R. chacei (Branchiostegite)*** |  |  | 12 | 8 |  | 12 | 4 to 6 |
| ***R. chacei (Scaphognathites)*** |  |  | 12 | 8 |  | 12 | 4 to 6 |
| ***R. chacei (Exopodites)*** |  |  | 12 | 8 |  | 12 | 4 to 6 |
| ***R. exoculata (cardiac chamber)*** | 2 | 4 | 4 | 4 | 4 | 4 | 4 to 6 |
| ***R. exoculata (pyloric chamber)*** | 2 | 4 | 2 | 4 | 4 | 7 | 4 to 6 |
| ***R. chacei (cardiac chamber)*** |  |  | 4 | 4 |  | 4 | 4 to 6 |
| ***R. chacei (pyloric chamber)*** |  |  | 4 | 4 |  | 4 | 4 to 6 |
| ***R. exoculata (midgut tube)*** | 2 | 4 | 6 | 4 | 4 | 8 | 5 to 8 |
| ***R. chacei (midgut tube)*** |  |  | 4 | 4 |  | 4 | 5 to 8 |

### Appendix 5:

**FISH robes used for this study.**

| **Target** | **Probe** | **Sequence (5’-3’)** | **Dye** | **Location (rRNA gene)** | **Optimal Formamide %** | **References** |
| --- | --- | --- | --- | --- | --- | --- |
| *Gammaproteo-bacteria* | GAM42a | GCCTTCCCACATCGTTT | cy3 / cy5 | 1027 (23S) | 30 | Manz et al., 1992 |
| *Campylobacterales* | Epsy549 | CAGTGATTCCGAGTAACG | ATTO488 / cy3 / cy5 | 549 (16S) | 30 | Lin et al., 2006 |
| *Bacteria* | Eub338 | GCTGCCTCCCGTAGGAGT | ATTO488 / cy3 / cy5 | 338 (16S) | 30 | Amann et al., 1990 |
| *Deferribacteres* | Def1229 | GCCCTCTGTATAGTCCATTG | cy3/cy5 | 1229 (16S) | 30 | Guéganton et al., 2022 |
| *Mycoplasmatales* | Myco378-1 | GTGGAAAATTCCCTACTGCTG | cy3 | 378 (16S) | 45 | Guéganton et al., 2022 |
| *Bacteriodia* | CF319a | TGGTCCGTGTCTCAGTAC | cy3 | 319 (16S) | 20 | Manz et al., 1996 |
| *Zetaproteobacteria* | Zeta709 | GCCTCAGGTGTTCCTCCG | cy3 | 709 | 20 | Hoshino et al., 2016 |
| *Desulfobulbales* | DSB706 | ACCGGTATTCCTCCCGAT | cy3/y5 | 706 | 35 | Lucker et al., 2007 |

### Appendix 6:

Nine samples (two foreguts, two midgut tubes, two cephalothorax and three gills) of *R. exoculata* juveniles (stage A) were amplified by both direct PCR and nested PCR, and were used to assess potential bias. Richness diversity analyses showed no influence of the PCR treatment (p = 0.8358) on sample diversity obtained, whereas the tissue significantly influenced diversity (p = 0.0053) according to Chao1 index (**Supplementary Table 1.**). The barplots of bacterial lineages composition relative abundance also visually confirmed that the same communities were retrieved whether the DNA was directly amplified or through nested PCR (**Supplementary Figure 1.**). We observed some slight differences but the overall lineage compositions were identical between direct PCR and nested PCR treatments. The results were statistically confirmed by ANCOM. No significant difference was observed at ASV, genus and family levels. (**Supplementary Table 2., Supplementary Table 3.**).

NMDS showed no clear separation of the sample between the direct and nested PCR with both amplifications of the same sample appearing very close to each other (**Supplementary Figure 1.**). β diversity analyses showed that PCR had no significant influence on bacterial diversity (PERMANOVA, p = 0.5621) contrary to the site and the tissues (p = 0.0001 both) (**Supplementary Table 1.**).

### Appendix 7:

Custom.config for the Nested and Direct PCR samples comparison

/*

* -------------------------------------------------

* Nextflow test config file for processes options

* -------------------------------------------------

* Defines general paths for input files and

* parameters for samba processes

*/

params {

// Analyzed project name

projectName = "Rimicaris_juvenile_nichee_simple_comparaison_juv_3_bis"

// Data type (paired by default)

singleEnd = false

// Output directory to publish workflow results

outdir = "/home1/datawork/mguegant/SAMBA-nextflow/JUVENILE/results/${projectName}"

// Input parameters for QIIME2

input_manifest = "/home1/datawork/mguegant/SAMBA-nextflow/JUVENILE/q2_manifest_PCR_Nichee_simple_comparaison_juv_bis.txt"

input_metadata = "/home1/datawork/mguegant/SAMBA-nextflow/JUVENILE/q2_metadata_PCR_Nichee_simple_comparaison_juv_bis.txt"

/*

Steps to activate or deactivate

*/

data_integrity_enable = true

cutadapt_enable = true

dbotu3_enable = true

filtering_tax_enable = false

microDecon_enable = true

picrust2_enable = true

ancom_enable = true

stats_alpha_enable = true

stats_beta_enable = true

stats_desc_comp_enable = true

report_enable = true

// To compress final result directory set compress_result to true

compress_result = true

//STATS ONLY : set stats_only to true if you already have your ASV table

stats_only = false

//Dada2 merge process

dada2merge = false

//Activate longreads for pacbio or nanopore input data (longreads=false by default to handle illumina short reads)

longreads = false

/*

Cleaning primers step using cutadapt

*/

//Set your forward and reverse primers for cutadapt

primerF = "CCTACGGGNGGCWGCAG"

primerR = "GACTACHVGGGTATCTAATCC"

//Error rate default=0.1

errorRate = "0.1"

//Overlap default=(length shortest primer - 1)

overlap = "16"

/*

ASV inference step using Dada2

*/

//adapt dada2 parameters to fit to your data

//trim default=0 (no trimming)

FtrimLeft = "0"

RtrimLeft = "0"

//trunclen : according to the quality plots of your data, set the length of the trimming (0=no initial trimming)

FtruncLen = "0"

RtruncLen = "0"

//MaxEE error rate allowed for reads ; default 2

FmaxEE = "2"

RmaxEE = "2"

//min. quality allowed ; default 2

minQ = "2"

//Chimeras : method used to remove chimeras ; choices: 'consensus' (default), 'none' or 'pooled'

chimeras = "consensus"

//to set if DADA2 MERGE ASVs tables is activated

//path to dada2 directory with ASV tables to merge (directory must contained only the tables to merge)

merge_tabledir = "/PATH/TO/merge/table"

//path to dada2 directory with repseqs to merge (directory must contained only the repseq files to merge)

merge_repseqsdir = "/PATH/TO/merge/seq"

/*

ASV taxonomic assignation using QIIME2 RDP-like program

*/

//pre-formatted databases can be download here: ftp://ftp.ifremer.fr/ifremer/dataref/bioinfo/sebimer/sequence-set/SAMBA/2019.10/

database = "/home/ref-bioinfo/ifremer/sebimer/tool-specific-banks/qiime2/2019.10/silva/v138/full_rRNA/silva_v138_16S-18S_99_full.qza"

//extract region from reference database using primers (default but takes time)

seqs_db = ""

taxo_db = ""

/*

Filtering ASV table and sequences based on the taxonomic assignation

*/

//list of taxa you want to exclude (comma separated list). Set to "none" if you choose to include taxa.

tax_to_exclude = ""

//list of taxa you want to include (comma separated list). Set to "none" if you choose to exclude taxa.

tax_to_include = ""

/*

Decontamination step using microDecon package

*/

//list of control samples. For one control: "control1" ; for more controls: "control1,control2". If no control, set to "none"

control_list = "Rem-T2,Rem-T2n1,Rem-T2n2,Rem-T2n3,Rem-T2n4,Rem-T2n5,Rem-Tn4"

//number of controls? If no control, set to "0"

nb_controls = "7"

//number of samples (total samples - number of controls)

nb_samples = "18"

/*

Differential abundance testing with ANCOM

*/

//Variables of interest (comma separated list)

ancom_var = "tissu,site,PCR,tissu_localisation"

/*

Functional predictions with PICRUSt2

*/

//Variables of interest (comma separated list)

picrust_var = "tissu,site,PCR,tissu_localisation"

/*

Remove samples for statistical analyses

*/

//Activate or desactivate this step in the create_phyloseq_obj.R process

remove_sample = false

//list of samples to remove (comma separated list)

sample_to_remove = ""

/*

Statistics steps parameters

*/

//According to your metadata file, list the column names corresponding to the variables of interest (comma separated list)

//Alpha diversity analysis

alpha_div_group = "tissu,site,PCR"

//Kingdom to be represented in barplots

kingdom = "Bacteria"

//Maximum number of taxa to represent

taxa_nb = "10"

//Beta diversity analysis

beta_div_var = "tissu,site,PCR"

//Hierarchical clustering method

hc_method = "ward.D2"

//UpsetR analysis (descriptive analysis like venn diagram)

desc_comp_crit = "tissu,site,PCR"

//taxonomic level for bar in UpsetR graph

desc_comp_tax_level = "Class"

//if STATS_ONLY is activated

inasv_table = "/PATH-TO/your_own_ASV_table.tsv"

innewick = "/PATH-TO/your_own_tree.nwk"

}

### Appendix 8:

Custom.config for all juvenile samples

/*

* -------------------------------------------------

* Nextflow test config file for processes options

* -------------------------------------------------

* Defines general paths for input files and

* parameters for samba processes

*/

params {

// Analyzed project name

projectName = "Rimicaris_juvenile_total_juv_sans_br_V4"

// Data type (paired by default)

singleEnd = false

// Output directory to publish workflow results

outdir = "/home1/datawork/mguegant/SAMBA-nextflow/JUVENILE/results/${projectName}"

// Input parameters for QIIME2

input_manifest = "/home1/datawork/mguegant/SAMBA-nextflow/JUVENILE/q2_manifest_total_juv_sans_br.txt"

input_metadata = "/home1/datawork/mguegant/SAMBA-nextflow/JUVENILE/q2_metadata_total_juv_sans_br.txt"

/*

Steps to activate or deactivate

*/

data_integrity_enable = true

cutadapt_enable = true

dbotu3_enable = true

filtering_tax_enable = false

microDecon_enable = true

picrust2_enable = true

ancom_enable = true

stats_alpha_enable = true

stats_beta_enable = true

stats_desc_comp_enable = true

report_enable = true

// To compress final result directory set compress_result to true

compress_result = true

//STATS ONLY : set stats_only to true if you already have your ASV table

stats_only = false

//Dada2 merge process

dada2merge = false

//Activate longreads for pacbio or nanopore input data (longreads=false by default to handle illumina short reads)

longreads = false

/*

Cleaning primers step using cutadapt

*/

//Set your forward and reverse primers for cutadapt

primerF = "CCTACGGGNGGCWGCAG"

primerR = "GACTACHVGGGTATCTAATCC"

//Error rate default=0.1

errorRate = "0.1"

//Overlap default=(length shortest primer - 1)

overlap = "16"

/*

ASV inference step using Dada2

*/

//adapt dada2 parameters to fit to your data

//trim default=0 (no trimming)

FtrimLeft = "0"

RtrimLeft = "0"

//trunclen : according to the quality plots of your data, set the length of the trimming (0=no initial trimming)

FtruncLen = "0"

RtruncLen = "0"

//MaxEE error rate allowed for reads ; default 2

FmaxEE = "2"

RmaxEE = "2"

//min. quality allowed ; default 2

minQ = "2"

//Chimeras : method used to remove chimeras ; choices: 'consensus' (default), 'none' or 'pooled'

chimeras = "consensus"

//to set if DADA2 MERGE ASVs tables is activated

//path to dada2 directory with ASV tables to merge (directory must contained only the tables to merge)

merge_tabledir = "/PATH/TO/merge/table"

//path to dada2 directory with repseqs to merge (directory must contained only the repseq files to merge)

merge_repseqsdir = "/PATH/TO/merge/seq"

/*

ASV taxonomic assignation using QIIME2 RDP-like program

*/

//pre-formatted databases can be download here: ftp://ftp.ifremer.fr/ifremer/dataref/bioinfo/sebimer/sequence-set/SAMBA/2019.10/

database = "/home/ref-bioinfo/ifremer/sebimer/tool-specific-banks/qiime2/2019.10/silva/v138/full_rRNA/silva_v138_16S-18S_99_full.qza"

//extract region from reference database using primers (default but takes time)

seqs_db = ""

taxo_db = ""

/*

Filtering ASV table and sequences based on the taxonomic assignation

*/

//list of taxa you want to exclude (comma separated list). Set to "none" if you choose to include taxa.

tax_to_exclude = ""

//list of taxa you want to include (comma separated list). Set to "none" if you choose to exclude taxa.

tax_to_include = ""

/*

Decontamination step using microDecon package

*/

//list of control samples. For one control: "control1" ; for more controls: "control1,control2". If no control, set to "none"

control_list = "Rem-T2n1,Rem-T2n2,Rem-T2n3,Rem-T2n4,Rem-T2n5,Rem-T2,Rem-Tn4"

//number of controls? If no control, set to "0"

nb_controls = "7"

//number of samples (total samples - number of controls)

nb_samples = "148"

/*

Differential abundance testing with ANCOM

*/

//Variables of interest (comma separated list)

ancom_var = "tissu,site,stage,species, PCR "

/*

Functional predictions with PICRUSt2

*/

//Variables of interest (comma separated list)

picrust_var = "tissu,site,stage,species,PCR

/*

Remove samples for statistical analyses

*/

//Activate or desactivate this step in the create_phyloseq_obj.R process

remove_sample = false

//list of samples to remove (comma separated list)

sample_to_remove = ""

/*

Statistics steps parameters

*/

//According to your metadata file, list the column names corresponding to the variables of interest (comma separated list)

//Alpha diversity analysis

alpha_div_group = "tissu,site,stage "

//Kingdom to be represented in barplots

kingdom = "Bacteria"

//Maximum number of taxa to represent

taxa_nb = "10"

//Beta diversity analysis

beta_div_var = "tissu,site,stage,species "

//Hierarchical clustering method

hc_method = "ward.D2"

//UpsetR analysis (descriptive analysis like venn diagram)

desc_comp_crit = "tissu,site,stage,species,,tissu_stage_species,site_species_stage"

//taxonomic level for bar in UpsetR graph

desc_comp_tax_level = "Class"

//if STATS_ONLY is activated

inasv_table = "/PATH-TO/your_own_ASV_table.tsv"

innewick = "/PATH-TO/your_own_tree.nwk"

}
